# Computational Models Accurately Predict Multi-Cell Biomarker Profiles in Inflammation and Cancer

**DOI:** 10.1038/s41598-019-47381-4

**Published:** 2019-07-26

**Authors:** Carol L. Fischer, Amber M. Bates, Emily A. Lanzel, Janet M. Guthmiller, Georgia K. Johnson, Neeraj Kumar Singh, Ansu Kumar, Robinson Vidva, Taher Abbasi, Shireen Vali, Xian Jin Xie, Erliang Zeng, Kim A. Brogden

**Affiliations:** 1 Department of Biology, Waldorf University, Forest City, IA 50436 USA; 2 Department of Human Oncology, University of Wisconsin School of Medicine and Public Health, University of Wisconsin-Madison, Madison, WI 53705 USA; 3 Department of Oral Pathology, Radiology and Medicine, College of Dentistry, University of Iowa, Iowa City, IA 52242 USA; 4 College of Dentistry, University of Nebraska Medical Center, Lincoln, NE 68583 USA; 5 Department of Periodontics, College of Dentistry, University of Iowa, Iowa City, IA 52242 USA; 6 Cellworks Group Inc., San Jose, CA 95110 USA; 7 Cellworks Research India Pvt. Ltd (Wholly owned subsidiary of Cellworks Group Inc.), Bangalore, India; 8 Division of Biostatistics and Computational Biology, College of Dentistry, University of Iowa, Iowa City, IA 52242 USA; 9 Iowa Institute for Oral Health Research, College of Dentistry, University of Iowa, Iowa City, IA 52242 USA

**Keywords:** Computational models, Multicellular systems, Predictive markers

## Abstract

Individual computational models of single myeloid, lymphoid, epithelial, and cancer cells were created and combined into multi-cell computational models and used to predict the collective chemokine, cytokine, and cellular biomarker profiles often seen in inflamed or cancerous tissues. Predicted chemokine and cytokine output profiles from multi-cell computational models of gingival epithelial keratinocytes (GE KER), dendritic cells (DC), and helper T lymphocytes (HTL) exposed to lipopolysaccharide (LPS) or synthetic triacylated lipopeptide (Pam3CSK4) as well as multi-cell computational models of multiple myeloma (MM) and DC were validated using the observed chemokine and cytokine responses from the same cell type combinations grown in laboratory multi-cell cultures with accuracy. Predicted and observed chemokine and cytokine responses of GE KER + DC + HTL exposed to LPS and Pam3CSK4 matched 75% (15/20, p = 0.02069) and 80% (16/20, P = 0.005909), respectively. Multi-cell computational models became ‘personalized’ when cell line-specific genomic data were included into simulations, again validated with the same cell lines grown in laboratory multi-cell cultures. Here, predicted and observed chemokine and cytokine responses of MM cells lines MM.1S and U266B1 matched 75% (3/4) and MM.1S and U266B1 inhibition of DC marker expression in co-culture matched 100% (6/6). Multi-cell computational models have the potential to identify approaches altering the predicted disease-associated output profiles, particularly as high throughput screening tools for anti-inflammatory or immuno-oncology treatments of inflamed multi-cellular tissues and the tumor microenvironment.

## Introduction

Future anti-inflammatory or immuno-oncology (I-O) therapeutics will need to consider the impact of multiple and varied cell-types, rather than the impact of individual cells, in treating inflamed multi-cellular tissues or the cancer microenvironment^1^. Cultivating live cells together in multi-cell cultures represents a contemporary advance towards these concepts^2,3^ (see culture models in Supplementary Table S1). Multi-cell cultures are an avenue to facilitate the understanding of cell physiology, cell functionality, and signaling networks among cells in a fashion that begins to resemble *in vivo* tissue responses. They can model microbial biofilm-to-cell interactions, cell-to-cancer cell interactions in the tumor environment, the effects of cell interactions on adjacent cell proliferation and immune cell migration, biomarker production, and the effects of drugs on cancer cell viabilities. Cells have been cultivated in liquid-based systems as heterotypic cultures of cells in spheroids, organoids, and tumoroids or in transwell co-cultures. Cells have also been co-cultivated on scaffold-based systems to assess bio-matrices that contain structural proteins and growth factors important in tissue organization (again see Supplementary Table S1) and some systems utilize organic bioelectronic devices to monitor real-time adhesion and growth of cells in 3D cell cultures^4^. However, challenges are recognized in both preparing and using these co-culture systems in a high throughput manner to rapidly, accurately, and consistently assess the effects of therapeutics on cells, their pathways, and their combined chemokine, cytokine, and cellular biomarker profiles.

Computational platforms represent a novel alternative approach to establishing and using both single cell cultures and multi-cell cultures in the laboratory. Computational platforms capable of modeling differing aspects of cell-cell interactions have recently appeared with intent to (i) interface with automated image systems to screen and select tumor spheroids or tumor tissues for analysis^5–7^, (ii) model intercellular signaling networks among cells to identify molecular mechanisms underlying inflammation-associated tumourigenesis^8,9^, and (iii) identify novel anti-inflammatory and anti-cancer targets^9^. In this study, we created and combined individual computational models of single myeloid, lymphoid, epithelial, and cancer cells together to form multi-cell computational models. We used these models to predict the collective chemokine, cytokine, and cellular biomarker profiles often seen in inflamed or cancer tissues. We validated their output responses against retrospective studies in the literature and in the same cell type combinations grown in laboratory multi-cell cultures with accuracy. Multi-cell computational models became ‘personalized’ when MM cell line-specific genomic data were included into simulations, again validated with the same cell lines grown in laboratory multi-cell cultures. Multi-cell computational models have the potential to identify approaches altering the predicted disease-associated output profiles, particularly as high throughput screening tools for anti-inflammatory or I-O treatments of inflamed multi-cellular tissues and the tumor microenvironment.

## Materials and Methods

### Computational model data acquisition

**Figure 1:**
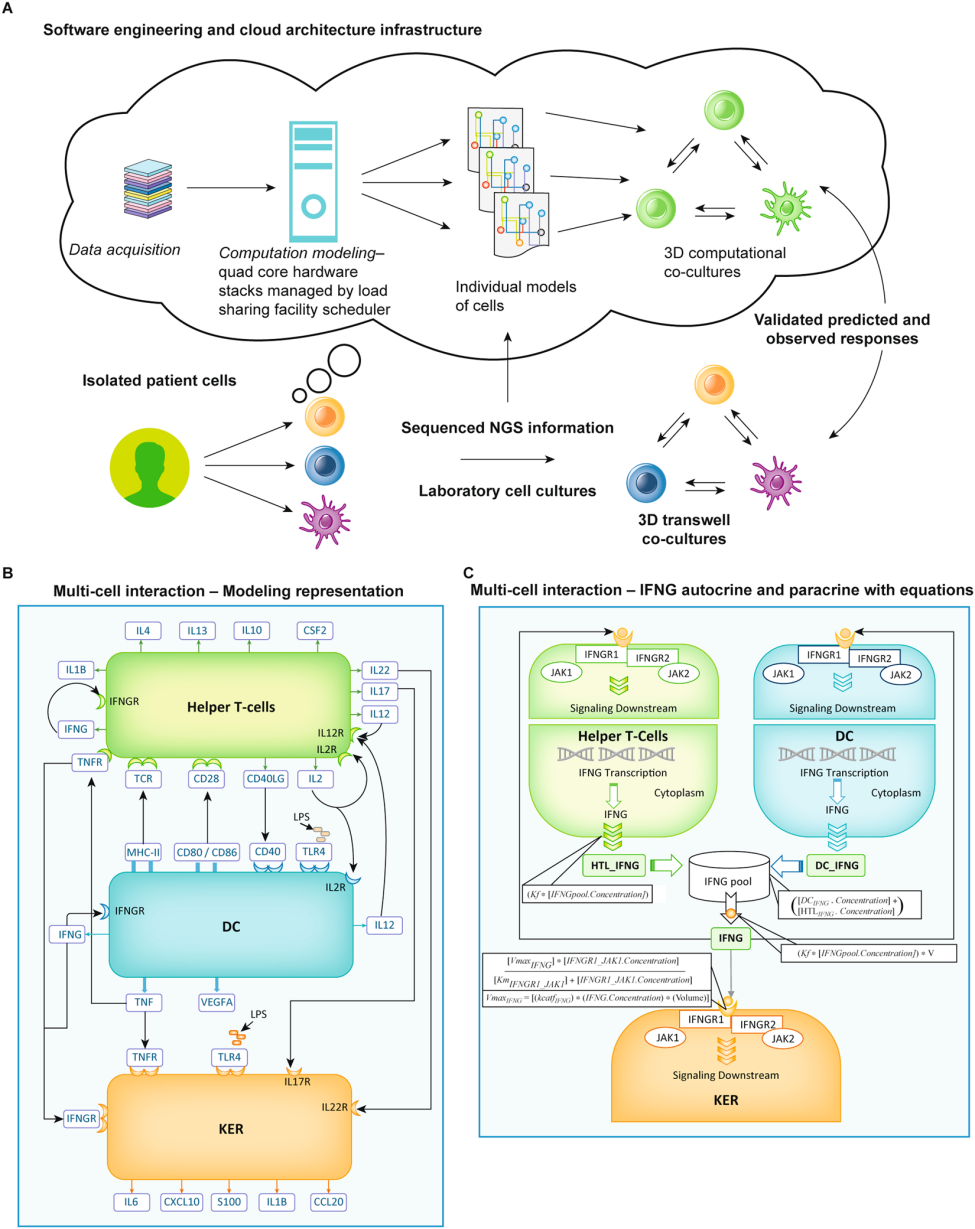
An illustration shows the general process for creating single cell and multi-cell computational models that were validated directly by comparing the predicted results against the chemokine and cytokine profiles produced from the same cells grown in single cell and multi-cell cultures. (**A**) Cell-specific computational models were created in a series of successive steps. Information from high quality genomic, transcriptomic, proteomic, and metabolomic datasets were downloaded into the computational network library and used to create computational models for single GE KER, DC, and HTL. We used these individual models to create GE KER + DC + HTL multi-cell computational models to predict the effects of inflammatory events. The predicted responses were validated against the observed responses of the same cells grown in multi-cell cultures. (**B**) In multi-cell cultures, each cell type reacts to both the presence of an added LPS agonist as well as the presence of chemokines and cytokines produced from adjacent cells, and vice versa. (**C**) The autocrine and paracrine responses of the multi-cell interaction were modeled and the IFNG pathway is included as an example. The predictive responses were calculated using Michaelis Menton kinetics; mass action kinetics; as ordinary differential equations (ODEs). ODEs contained the reaction; enzyme; initial concentrations of protein intermediate reactants; and the Ka, Km, kcat, and Vmax parameters of the reactants at each step of the signaling process.

We first identified general and cell type-specific information on cell signaling processes by searching the literature, supplementary databases, and data repositories for high quality genomic, transcriptomic, proteomic, and metabolomic datasets (Fig. 1). This information was reviewed and imported into the computational network library. This process was extensively described in a series of previous studies^10–12^. An example of this process was the dataset published by Rizvi *et al*. in *Science*^13^ that we used to affirm programmed death-ligand 1 (PD-L1) expression in patients with non-small cell lung cancers and predict their clinical responses to anti-programmed death-1 (PD-1) immunotherapy^12^.

We searched and included datasets on all aspects of the signaling process: signaling ligands and agonists; cellular receptors; signaling pathways, pathway intermediates, pathway regulators and feedback controls, cross-talk interactions among pathways, and pathway enzyme kinetics; and activation and regulation of transcription factors and production of gene products. We included datasets on intracellular processes such as proteasomal degradation, endoplasmic reticulum (ER) stress, oxidative stress, DNA damage and repair pathways, and cell cycle pathways. Finally, datasets were also imported important to many cellular pathways involved in cell adhesion, angiogenesis, cell death, cell autophagy, cell cycle, checkpoint control, cellular metabolism, epigenetics, cell cytoskeletal regulation, immunology, inflammation, MAP kinase signaling, PI3K/Akt signaling, cellular translational control, tyrosine kinase signaling, and ubiquitin signaling.

### Creation of computation models

**Figure 2:**
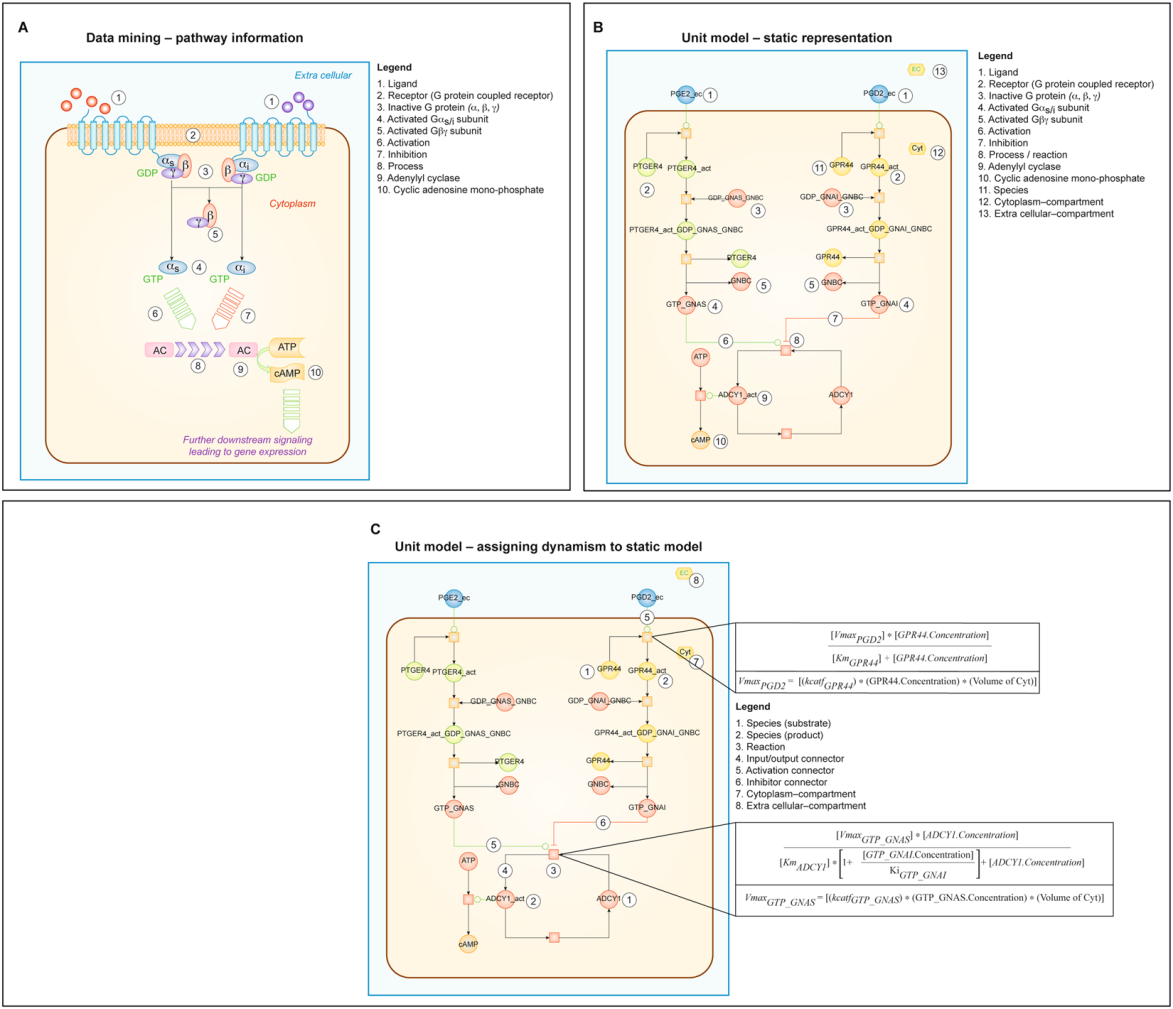
A schematic representation of a modeled signaling pathway using a G Protein Coupled Receptor activation as an example. (**A**) In an illustrated pathway, the ligand (1) binds to the G protein coupled receptor (2). The G protein (α,β,γ) exchanges GDP for GTP and dissociates into the Gα_s/i_ and the G_βγ_ subunits (3, 4, 5). The activated α_s_ unit activates (6) and the activated α_i_ subunit inhibits (7) adenylyl cyclase (8, 9) conversion of ATP to cAMP (10). We included PMID references that support each step throughout the pathway. (**B**) In a static representation of this pathway, the ligand PGE2 (1) binds to the G protein coupled receptor PTGER4 (2) in an activation pathway (green line) and PGE2 binds to GPR44 (11) in an inhibition pathway (red line). In the activation pathway, activated PTGER4_act, combines with inactive G protein (α,β,γ) (3) to form PTGER4act_GDP_GNAS_GNBC. PTGER4 and activated G_βγ_ subunit GNBC (5) dissociate. Activated Gα_s/i_ subunit (4) provides the activation signal (8) to adenlyl cyclase ADCY1 to ADCY1_act (9) for the conversion of ATP to cAMP (10). In an inhibition pathway, activated GPR44_act combines with the inactive G protein (α, β, γ) GDP_GNAI_GNBC (3) to form GPR44_act_GDP_GNAI_GNBC. GPR44 and activated G_βγ_ subunit GNBC (5) dissociate. Activated Gα_s/i_ subunit GTP_GNAI is inhibitory (7) to adenlyl cyclase ADCY1 to ADCY1_act (9) for the conversion of ATP to cAMP (10). (**C**) Dynamism is assigned to the static model at every step as illustrated for PGE2 binding to GPR44 (5) in the Activation Connector and at the activation and inhibition of the Reaction (3) of adenlyl cyclase ADCY1 to ADCY1_act (1, 2, 3) in the Input/Output Connector (4) per Michaelis Menton kinetic equations containing reactant concentrations and rate of reactions (Vmax). (**A**) Was adapted from *Molecular Biology of the Cell*, *3th Edition*. Copyright © 2015 by Bruce Alberts, Alexander Johnson, Julian Lewis, David Morgan, Martin Raff, Keith Roberts, and Peter Walter. Used with permission of the publisher, W. W. Norton & Company, Inc. All rights reserved.

We used the computational network library to create generic project-specific computational models for GE KER, DC, and HTL single cells (Fig. 1). Information on individual pathway nodes, their functionality, and their links with other genes, proteins, and pathways were identified in the library and then assembled into pathway-specific integrated network mazes. A graphical representation of such a cell-specific network (Fig. 2A) and the underlying network relationships (Fig. 2B,C) is illustrated.

We next used the computational network library to create cell line-specific computational models. We identified mutations and copy number variations for multiple myeloma (MM) cell lines MM.1S and U266B1 from next generation sequencing (NGS) information taken from the cBioPortal for Cancer Genomics database^14,15^, the TCGA Research Network (http://cancergenome.nih.gov/), and published information^16–18^. Mutated genes in MM.1S and U266B1 were compared to those in the Learnt Mutational Library. If the effect of gene mutation on gene function was in the Library, then that result was used in the model. If not, the effect of gene mutation on gene function was determined using the cancer mutation effect prediction algorithms FATHMM^19^, Mutation Assessor^20^, Polyphen^21^, PROVEAN^22^, and SIFT^23^. Final results were recorded as an effect of unknown signature, neutral to gene function, or deleterious to gene function. This information was then annotated into the generic MM models to create cell line-specific computational models for MM.1S and U266B1 similar to that in our previous study^10^.

At the network level, we represented mutations of oncogenes as gain of function at the activity level. We represented mutations of tumor suppressor genes as a loss of function at the activity level, unless explicit functionality of the mutation was known from published studies. We represented copy number variations, such as amplifications and deletions, as over-expression or deletion of gene function at the expression level.

### Determining computation model predictions

**Figure 3:**
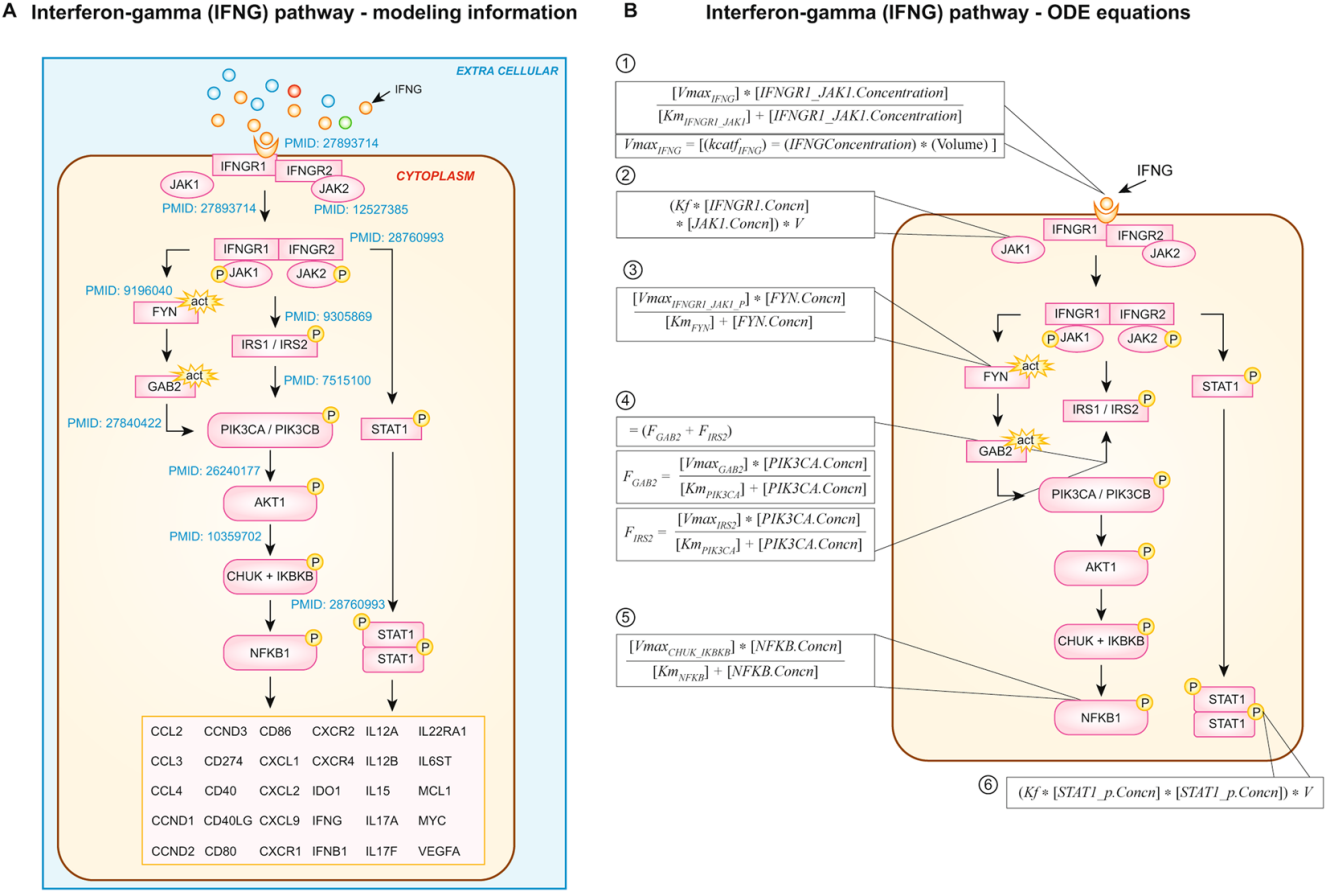
The interferon-gamma (IFNG) pathway and its modeled representation to demonstrate the step-by-step calculations for protein-protein interactions at each node in this pathway and as an annexure section of other pathways. The network was predictive and biomarker outputs were read as percent change in expression with respect to the control network. (**A**) In the IFNG pathway IFNγ binds with IFNGR1 (e.g., IFNGR alpha chain). IFNGR beta chain is IFNGR2. Downstream signaling requires Janus-activated kinase 1 (JAK1), JAK2 which mediate Signal Transducer and Activator of Transcription 1 (STAT-1) activation, thereby transcribing pro-inflammatory markers and immune-checkpoint markers. (**B**) We included PMID references that support each step throughout the pathway. In the modeled representation of the IFNG pathway, ligand IFNG binds to receptor represented by Simple Michalis Menten Equation (SMM) where the IFNG acts as an inducer for receptor activation (1). FYN activated by ligand (3) bound activated receptor complex (IFNGR + JAK complex), represented by SMM. PIK3CA activation by GAB2 and IRS2 (4), where flux would be driven by more than one activator, therefore each activator is represented by SMM and the final flux would be the summation of individual reactions. CHUK and IKBKB mediated NFKB phosphorylation (5) is represented by SMM, where CHUK-IKBKB complex phosphorylates cytosolic NFKB and trans-locates to nucleus for gene transcription. IFNGR1 binding to JAK1 (2) is represented as irreversible mass action equation and STAT1 dimerization (6) is represented by irreversible mass action equation.

Dynamism was assigned to the events in signaling pathways by calculating the pathway flux at each step in the signaling process (Fig. 2). Every process or reaction was modeled mathematically using Michaelis Menton kinetics, mass action kinetics, and variations of these representations using ordinary differential equations (ODEs)^24^ solved at each step by the Radau method^25^. Equations contained the reaction, enzyme, initial concentrations of protein intermediate reactants, and Ka, Km, kcat, and Vmax parameters of the reaction. These ODE models expressed the time-dependent concentration of a signaling molecule as a function of other molecules downstream and/or upstream within the pathway. This approach modeled protein-protein interactions at each step in a signaling pathway to predict specific pathway output^26^. To demonstrate this modeling approach, an annexure section of the PD-L1 pathway was illustrated in a recent study showing the step-by-step details of the protein-protein interactions at each node in the pathway as an example of the modeling process^12^. In this study, an annexure section of the IFNG pathway was illustrated also showing the step-by-step details of the protein-protein interactions at each node in the pathway that also occurred in all of the other pathways (Figs 3 and 3B).

### Software engineering platform

We used a software engineering and cloud architecture infrastructure (Fig. 1A). The physiology network and automation software can run millions of simulation studies in batch mode. The cloud architecture infrastructure consists of 150 quad-core hardware stacks managed by a software driven load sharing facility scheduler to manage the large simulation runs. The software engineering consisted of simulation stacks of the single cell computational models without and with cell line-specific genomic signature input.

### GE KER, DC, and HTL multi-cell inflammatory computational model

We prepared and used single cell computational models for gingival epithelial keratinocytes (GE KER), dendritic cells (DC), and helper T lymphocytes (HTL) as previously described^27–29^ and similar to those listed in Supplementary Fig. S1. We used these individual models to create GE KER + DC + HTL multi-cell computational models to predict the effects of inflammatory events. Connections integrated the common inputs and outputs representing paracrine and autocrine signaling between the cell types. Multi-cell computational models of inflammatory events were simulated by adding lipopolysaccharide (LPS) or synthetic triacylated lipopeptide (Pam3CSK4) into the simulation protocol and determining the output of downstream markers CSF2 (GM-CSF), CCL3 (MIP1α), CCL4 (MIP1β), CCL5 (RANTES), IL1α, IL6, IL8, TNFα, IL12(p40), and VEGF. Percent change in each output measure (with respect to the control network) was calculated after introduction of each agonist, and the magnitude and direction of these predictions were determined. This model represented the pro-inflammatory effect agonists can have on GE KER + DC + HTL marker expression.

### MM and DC multi-cell cancer computational model

We created ‘personalized’ predictive cancer models for MM cell line MM.1S (ATCC CRL-2974) and MM cell line U266B1 (ATCC TIB-196) as previously described^10,28–30^ and were similar to the models listed in Supplementary Fig. S1. Cell line-specific genomic data was annotated into MM computational models. Cell associated biomarkers CD47, FASL, and PD-L1 and cell-free chemokines and cytokines IL6, IL-10 TGFB1, and VEGFA responses were predicted. The predicted responses from MM.1S and U266B1 were compared to each other and to the observed responses from MM.1S and U266B1 grown in culture.

Computational models of U266B1 and MM.1S were combined with DC to create MM.1S + DC and U266B1 + DC multi-cell computational models to predict the ability of these cells to inhibit DC marker expression. Connections integrated the common inputs and outputs representing paracrine and autocrine signaling between the cell types. Percent change in each output measure (with respect to the control network) was calculated after considering the output of MM cell lines as input into the DC single cell computational model. The magnitude and direction of these predictions was determined. DC biomarker responses for CD80, CD86, IL2, IFNG, and IL12B were predicted. Match rates were measured to assess whether U266B1 or MM.1S inhibited DC marker expression more in co-culture.

### Agonists

*Escherichia coli* K12 lipopolysaccharide (LPS; 0.1, 1.0, and 10.0 µg/ml; InvivoGen, San Diego, CA) and Pam3CSK4 (0.1, 1.0, and 10.0 µg/ml; InvivoGen, San Diego, CA) were used as agonists to induce pro-inflammatory responses in single cell cultures and multi-cell cultures. Weight per volume stock solutions were prepared in pyrogen-free 0.01 M sodium phosphate with 0.140 M NaCl, pH 7.2 (PBS) containing 4.0 + 0.7 SEM (n = 3) pg/ml endotoxin (QCL-1000, Lonza Walkersville, Inc., Walkersville, MD). Stock solutions were then diluted in LGM-3 before use.

10.0 µg/ml *E*. *coli* K12 LPS (InvivoGen, San Diego, CA) and 10.0 µg/ml Pam3CSK4 (InvivoGen, San Diego, CA) were selected as optimum doses for each agonist and used to induce pro-inflammatory events in both the multi-cell computational models and multi-cell cultures.

### Cell lines

Normal human epidermal KER (NHEK 22179, Lonza Walkersville, Inc., Walkersville, MD) and primary gingival epithelial (GE) KER^31^ were used in preliminary experiments. Although the skin KER were more responsive to agonist treatments, GE KER more closely matched predictive responses of our simulation model (data not shown); therefore, we chose to utilize GE KER for these studies. GE KER were isolated as previously described^31^ from healthy gingival tissue samples obtained from healthy non-smoking individuals who underwent crown lengthening or canine exposure procedures. Informed consent was obtained from these individuals per a reviewed and approved protocol from the University of Iowa Institutional Review Board for the Use of Human Subjects in Research (number 199811030, November 6, 2005 to August 25, 2012). All methods were performed in accordance with the relevant guidelines and regulations. Primary, first passage cells GE375 was used.

GE KER identity was determined by immunohistochemistry (University of Iowa Diagnostic Laboratories, Iowa City, IA) using antibodies to vimentin (Dako, Carpenteria, CA; dilution 1:900) and pancytokeratin (AE1/AE3, dilution 1:200; cytokeratin 7, dilution 1:100; cytokeratin 8/18, dilution 1:200; Dako, Carpenteria, CA).

GE KER identity was confirmed by standard flow cytometry techniques using antibodies to intracellular (cytokeratins 14, 15, 16, 19, BD Pharmingen, San Jose, CA and cytokeratins 4, 5, 6, 8, 10, 13, 18, Cell Signaling, Danvers, MA), and surface KER markers (cytokeratin CD24, BD Pharmingen, San Jose, CA and CD104, BioLegend, San Diego, CA) (data not shown). Flow cytometry was completed at the University of Iowa Flow Cytometry Core Facility utilizing an LSR II Flow Cytometer. Prior to use in co-culture experiments, GE KER were suspended at 1 × 10^5^ viable cells/ml in Lymphocyte Growth Medium-3 (LGM-3, Lonza Walkersville, Inc., Walkersville, MD).

Human monocyte-derived immature myeloid DC (DC, AllCells, Alameda, CA) were positively identified by flow cytometry and contained DC of myeloid origin: 31.5% CD1c^+^/79.1% CD11c^+^ cells and 54.4% CD141^+^/79.1% CD11c^+^ cells (human myeloid DC multi-color flow cytometry kit FMC016, R&D Systems, Inc., Minneapolis, MN). DC were suspended at 1 × 10^5^ viable cells/ml in LGM-3.

Normal, non-activated peripheral blood CD4^+^ HTL (StemCell Technologies, Inc., Vancouver, BC Canada) were utilized for preliminary experiments; however, non-activated HTL showed a lesser response to treatment with agonists. Activated HTL were more responsive and the responses more closely matched predicted responses of our computational model (data not shown). Therefore, HTL were cultured in TexMACS medium (Miltenyi Biotec, Auburn, CA) and activated using a HTL Activation/Expansion Kit (MACS, Miltenyi Biotec, Auburn, CA). Activated HTL were suspended at 1 × 10^5^ viable cells/ml in LGM-3.

MM cell lines MM.1S (ATCC CRL-2974, American Type Culture Collection, Manassas, VA) and U266B1 (ATCC TIB-196, American Type Culture Collection, Manassas, VA) were cultured in RPMI 1640 (ATCC, American Type Culture Collection, Manassas, VA) with 1% penicillin/streptomycin and either 10% (MM.1S) or 15% (U266B1) fetal bovine serum (ATCC, American Type Culture Collection, Manassas, VA)^10^. Prior to use in multi-cell cultures, cells were pelleted by centrifugation (Eppendorf 5801R, 400 rcf) for ten minutes at 4 °C and suspended at 1 × 10^5^ viable cells/ml in LGM-3 with 1% penicillin/streptomycin only, to minimize the effects of media on cellular production of cytokines and chemokines.

### GE KER, DC, and HTL multi-cell inflammatory culture

The inserts from a 24-well transwell plate (no. 3414, Corning, Inc., Corning, NY) were put into the inserts from a 12-well transwell plate (no. 3401, Corning, Inc., Corning, NY) and both inserts were put into the 12-well transwell plate bottom. 200 µl of DC culture was added to 800 µl LGM-3 and put into the bottom well. 200 µl of GE KER (GE375) were added to 300 µl LGM-3 and put into the middle 12 mm transwell insert. 200 µl of activated HTL were put into the top 6 mm transwell insert. Single cell cultures and multi-two cell cultures were used as controls and were placed in the same well configurations as that in the multi-three cell cultures. Laboratory multi-cell cultures were incubated at 37 °C in 5% CO_2_ for 2 hours before adding appropriate agonists.

10.0 µg/ml *E*. *coli* K12 LPS (InvivoGen, San Diego, CA) and 10.0 µg/ml Pam3CSK4 (InvivoGen, San Diego, CA), and PBS were added to the middle transwell inserts and plates were incubated at 37 °C in 5% CO_2_ for 64 hours.

### MM and DC multi-cell cancer culture

The inserts from a 12-well transwell plate (no. 3401, Corning, Inc., Corning, NY) were put into the 12-well transwell plate bottom. 200 µl of DC culture was added to 800 µl LGM-3 and put into the bottom well. 200 µl of MM cell lines, MM.1S or U266B1 were added to 500 µl LGM-3 and put into the 12 mm transwell insert. Single cell cultures were used as controls and were placed in the same well configurations as that in the multi-two cell cultures. Multi-cell cultures were incubated at 37 °C in 5% CO_2_ for 64 hours.

### Chemokine and cytokine analysis

At 16, 32, and 64 hours post-agonist exposure, 200 µl was removed from GE KER, DC, and HTL single cell cultures and GE KER, DC, and HTL multi-cell cultures. 200 µl of LGM-3 was added back. At 64 hours, cell culture media was removed from each transwell layer, filtered, and frozen at −80 °C until analysis. CCL3 (MIP1α), CCL4 (MIP1β), CCL5 (RANTES), CSF2 (GM-CSF), IL12(p40), IL1α, IL6, IL8, TNFα, and VEGF concentrations were determined using Milliplex immunoassays (Millipore, Billerica, MA, USA) routinely run in our laboratory^28,29^.

At 64 hours post incubation, all cell culture media was removed from the MM and DC multi-cell culture, filtered, and frozen at −80 °C until analysis. MM cell lines and DC were collected and lysed. Concentrations of IL6, IL10, VEGF, and TGFβ1 were determined in the cell culture media using Milliplex immunoassays (Millipore, Billerica, MA, USA) and concentrations of PD-L1, CD47, IDO, and FASL in cell lysates were determined using enzyme-linked immunosorbent assay kits (ELISA, American Research Products, Inc., Waltham, MA).

Standard curves for each cytokine were prepared from 0.64 to 10,000.00 pg/ml and concentrations of chemokines and cytokines in each sample were interpolated from standard curves (MILLIPLEX Analyst v5.1, Millipore, Billerica, MA or xPonent v3.1, Luminex, Austin, TX).

### Validation and statistical analysis

The integrity of the computational network, the accuracy of single cell computational models, the accuracy of multi-cell computational models, and the precision of the model output readouts were all checked and validated.

The reliability and integrity of modelled pathway information in the network was validated using internal control analysis checks to monitor the effects of pathway molecule over-expression and molecule knockdown on pathway predictions; effects of drugs on pathway predictions; and the activation, regulation, and cross-talk interactions among pathway intermediates on pathway predictions. BIRC5, an inhibitor of apoptosis, is selectively overexpressed in common human cancers and was used as an example of this validation process. In the computational network library, BIRC5 was listed in 126 studies.

The accuracy of single cell computational models was also validated in retrospective studies by comparing the predicted model results against retrospective experimental results of published laboratory and clinical trial results identified in the literature. Examples of these types of validation are shown in Supplementary Table S2 and Supplementary Table S3.

The accuracy of single cell computational models and multi-cell computational models were validated directly by comparing the predicted results against the chemokine and cytokine profiles of the same cells grown in single cell and multi-cell cultures. The trend comparison among biomarkers were tabulated as binary outcomes (“match” vs “mismatch”) and, for example, the matching result of 9 out of 10 biomarker matches gives an exact p-value of 0.01 using one-sided cumulative binomial distribution assuming complete randomness (null matching probability of 0.5).

## Results

In our prior work, we successfully created single cell computational models to predict cell biomarker profiles (Supplementary Fig. S1). Our ‘toolbox’ contained cell type-specific computational models of over 18 different cell types. There were individual computational models of non-immune cells (e.g., epithelial cells, stromal cells, myometrial cells, dopaminergic cells, keratinocytes, and human dermal fibroblasts); immune cells (e.g., DC, HTL, and normal primary bone marrow CD34^+^ cells); and cancer cells (e.g., human hepatocellular carcinoma cells, gastric cancer cells, glioblastoma, MM cells, and head and neck squamous cell carcinoma cells). In the present study, we recreated single cell computational models of GE KER, DC, HTL, and MM cell lines MM.1S and U266B1. Individual computational models were created using information ‘mined’ from cell-type specific studies containing genomic, transcriptomic, proteomic, and metabolomic datasets as recently described^10–12,30,32,33^.

Time-dependent changes in signaling pathway fluxes were represented via mass action or Michaelis Menton kinetic equation formalisms determined utilizing algorithms and modified ODEs^10,12,30^. For example, Fig. 2 illustrates this process using a schematic representation of a modeled signaling pathway for G Protein Coupled Receptor activation. Figure 2A illustrates a descriptive representation of this pathway and Fig. 2B shows the actual activation (green line) and inhibition (red line) pathways for the conversion of ATP to cAMP. Dynamism was assigned to the static model (Fig. 2C). Even though only two reactions are shown, there were reactions for every step in that schematic represented by the squares. As our example in Fig. 2C, PGE2 binds to GPR44 in the Activation Connector and at the activation and inhibition of the Reaction of adenlyl cyclase ADCY1 to ADCY1_act (activated adenylate cyclase 1) in the Input/Output Connector per Michaelis Menton kinetic equations containing reactant concentrations and rate of reactions (Vmax).

Kinetic parameters in the computational model were determined utilizing a semiconductor regression based automated modeling methodology. Machine learning techniques were used at the level of small biochemical pathways to understand their behavior and were represented mathematically in the model. Smaller pathways were combined into larger macro interactions using a manual curation approach. Information was aggregated through manual scientific review to maintain a high quality of input and to address issues of prevalent contradictory data points. An annexure section of the PD-L1 pathway was previously reported to demonstrate the step-by-step calculations for protein-protein interactions at each node in this pathway and for all of the other pathways^12,30^. An annexure section of the interferon-gamma (IFNG) pathway and its modeled representation is shown in Fig. 3A to demonstrate the step-by-step calculations (Fig. 3B) for protein-protein interactions at each node in this pathway.

Multi-cell computational models were then assembled from select individual computational models (Fig. 1) by inter-cellular signal transduction events (Fig. 1C). The platform running multi-cell computational models consisted of a bio-simulator with a cloud architecture infrastructure built to solve differential equations and other mathematical structures, at the rate of millions per second per bio-simulation.

### Validation of network, computational models, and model output

Internal control analysis checks were made on all genes and proteins in the network. The mention of survivin is only one example of the extensive control analysis checks in the system of validation. In this example, network predictions showed that a) over-expression and knockdown of activators and inhibitors altered BIRC5 expression; b) rapamycin-mediated mTOR inhibition attenuated survivin and inhibition of ErbB2 by herceptin reduced survivin expression; c) hypoxia-inducible factor 1α (HIF1α) mediated activation of survivin; d) cross-talk between EGFR and HIF1α up-regulated survivin, e) checks on the Bcr-Abl/MAPK cascade showed that BIRC5/TGFB/mTOR cross axis in IGF-1 mediated growth, f) BIRC5 was a novel target of Hedgehog/GLI1 signaling pathway, and g) activation of STAT3 was associated with elevated BIRC5 expression.

The computational model outputs were validated in retrospective studies by comparing the predicted results against retrospective experimental results of published laboratory studies and clinical trials identified in the literature. The dendritic cell computational model^28,29^ was extensively validated against published laboratory studies as shown in Supplementary Table S2 and a comparison of the observed reported responses and predicted responses of dendritic cells treated with LPS and pro-inflammatory agonists in 9 cocktail mixtures^34^ was shown in Supplementary Table S3.

**Figure 4:**
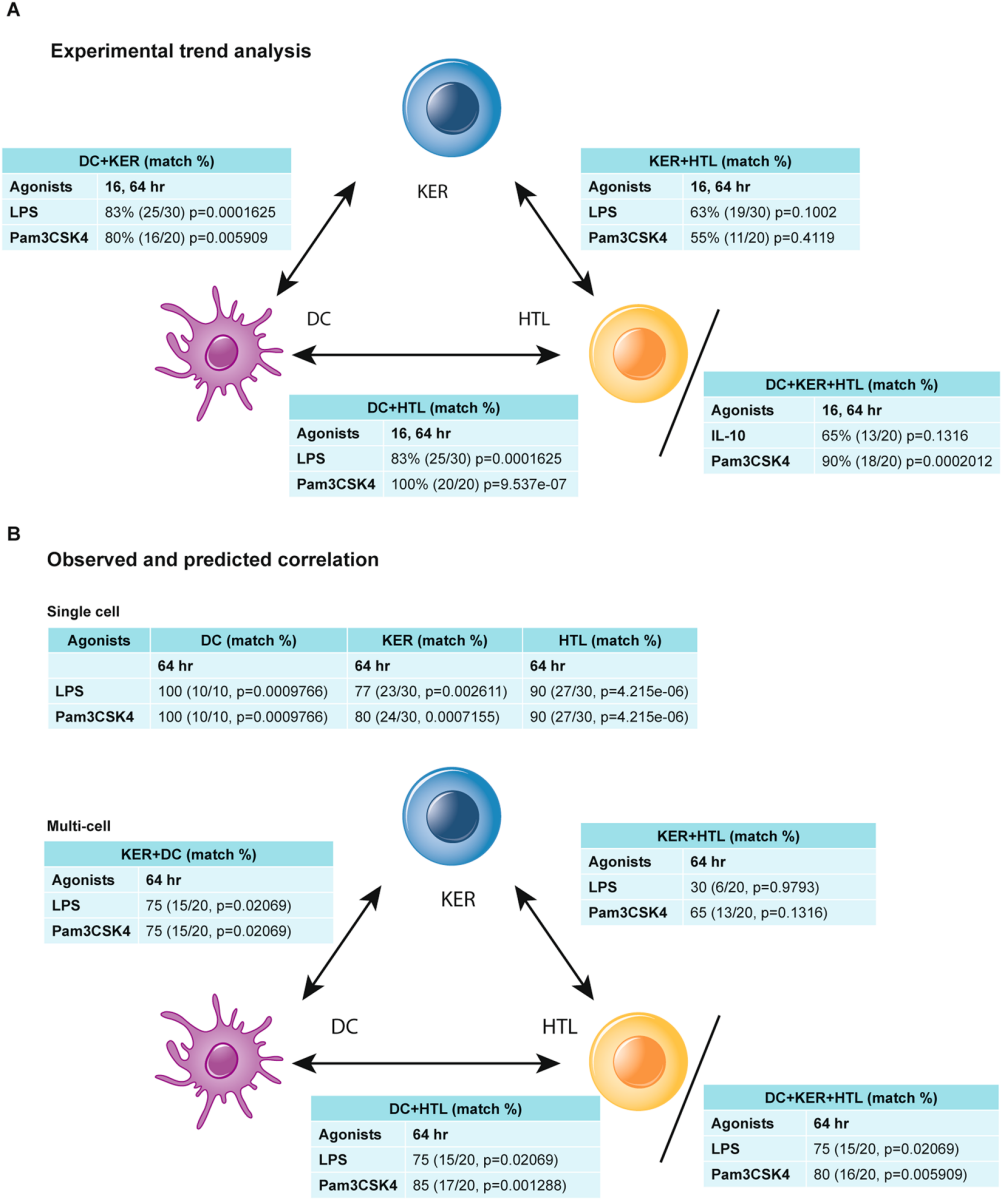
Comparison of match rates of predicted chemokine and cytokine responses for DC, KER, and HTL multi-cell computational model outputs versus observed chemokine and cytokine responses at 16 and 64 hours for the same cells grown in multi-cell culture. (**A**) Observed chemokine and cytokine responses of multi-cell cultures determining the trends for the biomarkers for the output of the individual cells. These results were based purely on the experimental trends. The observed responses were not compared to predictive responses. (**B**) Predictive vs. experimental data for responses to lipopolysaccharide (LPS) or synthetic triacylated lipopeptide (Pam3CSK4). Included are the percent correlations, the number positive matches/total, and the p values among the predictive and experimental datasets at 16 and 64 hours.

The model outputs were validated directly by comparing the predicted results against the chemokine and cytokine profiles of the same cells grown in single cell and multi-cell cultures. The network was predictive and chemokine and cytokine outputs were read as percent change in expression with respect to the control network. Chemokine and cytokine concentrations (pg/ml) from single cell and multi-cell cultures were determined and converted to percentage increase or decrease (with respect to cell culture controls)(Fig. 4). The trend comparison of increase or decrease in expression among predicted and observed responses were tabulated as binary outcomes (“match” vs “mismatch”) and, for example, the matching result of 9 out of 10 biomarker correlation gives an exact p-value of 0.01 using one-sided cumulative binomial distribution assuming complete randomness (null matching probability of 0.5).

### Creation and validation of GE KER, DC, and HTL multi-cell inflammatory computational model

In our first example, we modelled an agonist-induced pro-inflammatory event and measured chemokine and cytokine outputs from cells often involved in oral inflammation^35,36^. Optimum agonist doses were determined by exposing single cell cultures of GE KER, DC, and HTL to *E*. *coli* LPS and Pam3CSK4 at 3 concentrations each. We performed an Experimental Trend Analysis on the observed CSF2 (GM-CSF), CCL3 (MIP1α), CSF2 (GM-CSF), CCL5 (RANTES), IL1α, IL6, IL8, TNFα, IL12(p40), and VEGF responses calculated with respect to (wrt) controls (Fig. 4A). This was done by determining the effect each single cell culture response had to the overall trends of the two cell culture and the three cell culture responses. Responses were recorded as the same (e.g., both single cell culture responses increased or decreased wrt controls) or opposite (e.g., a single cell culture response increased and the other one decreased wrt controls). The expected single cell trend similarity values were summed, compared to the actual response observed for the two cell culture and the three cell culture responses, and recorded as match or mismatch percent (Fig. 4A). Values of 2 and 3 replications in the 16 and 64 hour data were pooled resulting in 20–30 events to be matched. There were significant (p < 0.05) matches of responses with both the pro-inflammatory agonists LPS and Pam3CSK4.

We then assessed the correlation between the single cell and multi-cell culture trends and the predicted single cell computational and multi-cell computational model trends by matching the CSF2 (GM-CSF), CCL3 (MIP1α), CSF2 (GM-CSF), CCL5 (RANTES), IL1α, IL6, IL8, TNFα, IL12(p40), and VEGF responses (Fig. 4B, Supplementary Table S4). Match rates among single cell computational model outputs and single cell culture chemokine and cytokine profiles ranged from 77% (23/30, p = 0.002611 for KER exposed to 10.0 µg/ml *E*. *coli* K12 LPS) to 100% (10/10, p = 0.0009766 for DC exposed to 10.0 µg/ml *E*. *coli* K12 LPS and to 10.0 µg/ml Pam3CSK4)(Fig. 4B). Match rates among two cell computational model outputs and two cell culture chemokine and cytokine profiles ranged from 30% (6/20, p = 0.9793 for KER + HTL exposed to 10.0 µg/ml *E*. *coli* K12 LPS) to 85% (17/20, p = 0.001288 for DC + HTL exposed to 10.0 µg/ml Pam3CSK4). Match rates among three cell computational model outputs and three cell culture chemokine and cytokine profiles ranged from 75% (15/20, p = 0.02069 for DC + KER + HTL exposed to 10.0 µg/ml *E*. *coli* K12 LPS) to 80% (16/20, p = 0.005909 for DC + KER + HTL exposed to 10.0 µg/ml Pam3CSK4).

Looking closer at the pro-inflammatory *E*. *coli* LPS responses, single cell computational models and single cell cultures had match rates ranging from 80–90% (Supplementary Figs S2, S3, and Supplementary Table S4). Two cell computational models and two cell cultures had match rates at 80% (Supplementary Table S4). One mismatch occurred in the DC + HTL CCL5 (RANTES) response, two mismatches occurred in GE KER + DC and GE KER + HTL IL12(p40) responses, one mismatch occurred in DC + HTL IL1α response, and 2 mismatches occurred in GE KER + DC and GE KER + HTL VEGFA responses. A match rate of 90% was seen between the three cell computational model output and three cell culture chemokine and cytokine profile (Table 1). One mismatch occurred in the GE KER + DC + HTL CCL5 (RANTES) predicted and observed responses.

**Table 1:** The LPS-induced chemokine and cytokine responses were modelled and the outputs were measured.

Marker	Single cell culture						Three cell culture				
	GE		DC		HTL		Expected Magnitude* (sum)	Trend	Experimental Magnitude* (mean)	Predicted Magnitude*	Trend Comparison**
	Exp. Mag.* (mean)	Trend	Exp. Mag.* (mean)	Trend	Exp. Mag.* (mean)	Trend					
CCL3	155.31	▲	68.78	▲	40.38	▲	183.63	▲	30.79	155.31	MATCH
CCL4	172.51	▼	42.93	▲	34.27	▲	70.50	▲	36.79	172.51	MATCH
CCL5	201.01	▲	−14.13	▼	11.15	▲	5.44	▲	−7.95	201.01	**MISMATCH**
CSF2	195.28	▲	175.34	▲	−1.58	▬	186.12	▲	78.59	195.28	MATCH
IL12B	218.62	▲	−12.18	▼	−40.88	▼	−36.30	▼	38.42	218.62	MATCH
IL1A	244.17	▼	18.22	▲	3.39	▬	10.30	▲	33.60	244.17	MATCH
IL6	158.26	▲	72.64	▲	108.70	▲	212.62	▲	153.70	158.26	MATCH
IL8	254.02	▲	329.84	▲	197.74	▲	621.85	▲	178.15	254.02	MATCH
TNF	394.64	▬	64.46	▲	49.57	▲	114.94	▲	54.24	394.64	MATCH
VEGFA	222.71	▼	49.56	▲	1.48	▬	−31.56	▼	125.13	222.71	MATCH
										**Match %**	**90%**

The magnitude of the predicted and observed responses from individual single cells (columns 1–3) and 3 cell multiscale computational models (column 4) were compared. The experimental magnitude was the actual measurements of chemokines and cytokines with respect to controls at 64 hours. The expected magnitude is the sum of the magnitudes for each cell type when cultured individually with LPS with analytes measured at 64 hours. When compared, the observed and predicted responses matched with 9 out of 10 markers (p-value of the Binomial Test was 0.02148).

*Percent change with respect to control.

**Represents the match (or mismatch) between experimental versus predictive trend (e.g. direction of increase/decrease).

### Creation and validation of MM and DC multi-cell cancer computational model

**Figure 5:**
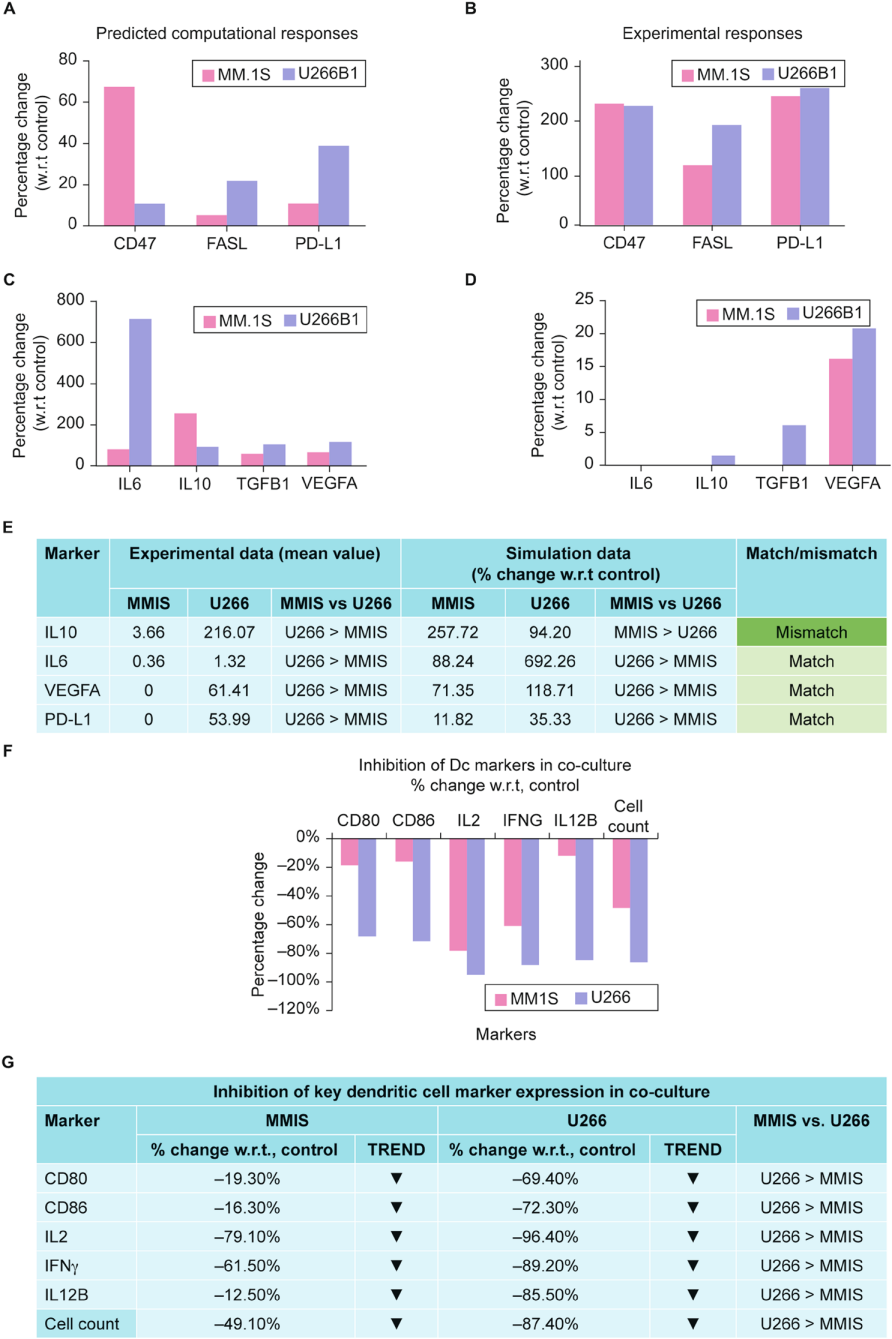
Personalized predictive cancer models were created for MM cell line MM.1S (ATCC CRL-2974) and MM cell line U266B1 (ATCC TIB-196). Single cell and multi-cell computational models became ‘personalized’ when cell line-specific genomic data were annotated into MM computational models and validated when predicted responses were compared to chemokine, cytokine, and cell-associated biomarker responses from the same cell lines grown alone or with DC in multi-cell cultures. (**A**–**D**) There were differences in the predicted and observed CD47, FASL, and PD-L1 responses and the IL6, IL-10 TGFB1, and VEGFA responses between MM.1S and U266B1. (**E**) When ‘personalized,’ U266B1 was predicted to have higher concentrations of IL-10, IL6, VEGFA, and PD-L1 than MM.1S and these predictions were validated by measuring the IL-10, IL6, VEGFA, and PD-L1 concentrations from MM.1S and U266B1 grown in single cell cultures. When the MM.1S vs. U266B1 responses were compared, the U266B1 > MM.1S matched 75% (3/4). (**F**) In multi-cell computational models with DC, U266B1 and MM.1S were predicted to inhibit DC CD80, CD86, IL2, IFNG, and IL12B responses. The percent change with respect to control was greatest with U266B1 > MM.1S. (**G**) Production of DC markers CD80, CD86, IL2, IFNG, and IL12B were lower when DC were cultured in multi-cell cultures with U266B1 or MM.1S: U266B1 attenuated DC marker production more than MM.1S and these responses matched 100% (6/6).

In our second example, we used individual models of MM.1S and U266B1 to predict the effects of cell line genomic signatures on their chemokine and cytokine outputs (Fig. 5). We then used these individual models to create MM.1S + DC and U266B1 + DC multi-cell computational models to predict the immunosuppressive effect cancer cells have on dendritic cell marker expression (Fig. 5).

U266B1 was predicted to have higher outputs than MM.1S (Fig. 5. Similarly, U266B1 was predicted to inhibit DC biomarkers (Fig. 5). Production of DC markers CD80, CD86, IL2, IFNG, and IL12B were lower when cells were grown with MM.1S or U266B1: U266B1 attenuated more DC marker production than MM.1S (Supplementary Fig. S3). To further test this, MM and DC multi-cell cancer computational models were created to assess the predicted effect of 23 myeloma cell lines on 19 DC biomarker readout responses. Supplementary Fig. S4 responses show differing effects MM cells have on DC chemokines and cytokines. High (green), moderate (yellow), and low (red) expression levels were predicted and the colors in this dataset represent demonstrate the vast effects of MM cells on dendritic cell biomarker readouts. The specific numbers indicate the percent changes from control baseline, respectively.

## Discussion

We report that multi-cell computational models can be created and used to predict collective biomarker profiles in a fashion that begins to resemble *in vivo* tissues. We created computational models of epithelial (GE KER), myeloid (DC), and lymphoid (CD4^+^ HTL) cells and combined them into multi-cell computational simulation models to predict the collective chemokine, cytokine, and cellular biomarker profiles in inflamed tissues (Fig. 1). We also created computational models of cancer (MM) cells and myeloid (DC) and combined them into multi-cell computational models to predict the chemokine, cytokine, and cellular biomarker profiles often inducing immunosuppressive effects on immune cells.

Many other combinations of multi-cell models can be created from the computational models in the ‘toolbox,’ capable of simulating events in inflamed multi-cellular tissues or the tumor microenvironment. The potential of this approach was illustrated in presented work using a multi-cell computational model of 8 cell types involved in the pathophysiology of rheumatoid arthritis^37^. Furthermore, these models became ‘personalized’ when cell line-specific genomic data were included into simulations^12,30^. Therapeutically, they have the potential to predict disease-associated output biomarker profiles and to identify approaches that return altered disease-associated output biomarker profiles to within normal limits. Thus, multi-cell computational models have the potential to become a high throughput screening tool to assess the dampening effects of new anti-inflammatory or I-O treatment drugs on disease-associated output biomarker profiles or alternately a tool to predict anti-inflammatory or I-O treatment options on cells of patients with these conditions.

At the cellular level, single cell computational models predict the molecular interactions and intracellular events noted above^10–12,30,32,33,38^. At the multi-cell level, models begin to predict the cell-cell interactions and collective chemokine, cytokine, and cellular biomarker profiles of cells in inflamed or cancer tissues^38,39^ (Fig. 1). This is an emerging field and there are other studies describing multi-cell models of such interactions^38,39^. One such model was constructed utilizing trigger-inducible gene switches in input-programmable circuits that enabled engineered cells to perform arithmetic calculations reminiscent of electronic circuits^40^. Different cell populations, each with genetic programs, encoded defined computational instructions and in 3D cultures executed programmable multicellular full-adder logics in response to trigger compounds.

Here, we presented 2 examples of an approach to predict the chemokine, cytokine, and cellular biomarker profiles to demonstrate that it is possible to combine single cell computational models to simulate cell-cell interactions likely occurring in inflamed multi-cellular tissues or the tumor microenvironment. The first example represented a model of inflammation containing KER, DC, and CD4^+^ HTL ‘triggered’ by exposure to pro-inflammatory agonists. Multi-cell computational models of these cells were created and stimulated with 2 agonists. The output of 10 chemokines, cytokines, and biomarkers were predicted and validated with cells grown in multi-cell cultures. The second example represented a model where MM cells induced immunosuppression on DC ‘triggered’ by differences in the genomic mutational signatures of each MM cell line. Each MM cell line had different predicted effects on the production of DC markers.

Both approaches have implications as high throughput screening tools to identify personalized therapeutics to lower biomarker profiles contributing to pro-inflammatory and immunosuppressive events in multi-cellular tissues and the cancer microenvironment. As more complex models evolve and contain genomic mutations, predictions will become more accurate with higher match rate percent validations. We also anticipate that such models will be able to predict anti-inflammatory or I-O treatment responses based on the genomics of the disease sample, that will attenuate predicted chemokine, pro-inflammatory cytokine, and immunosuppressive cellular biomarker output profiles, an application applicable to high throughput screening of therapeutic agents in multi-cellular tissues and the cancer microenvironment^30,32^. Furthermore, these methods can amplify the prediction of response to checkpoint inhibitors^12^ and also identify the right patient cohorts for CAR-T therapy response based on prediction of surface antigen expression and the immune microenvironment, an upcoming area of research focus, another emerging field.

Finally, computational platforms would avoid a trial and error style of approach and allow for the screening of therapies and the selection of promising combinations before going into clinical trials in the relevant patient segments. Such models would have the potential to identify treatment responders^12,30^, reduce the costs associated with inappropriate treatment, and reduce unnecessary medication side effects like that seen with some I-O treatments^41–45^.

## Supplementary information

Supplementary Dataset 1
