# Supplementary material for: Computational Models Accurately Predict Multi-Cell Biomarker Profiles in Inflammation and Cancer
Source: Sci Rep. 2019 Jul 26;9:10877. doi: 10.1038/s41598-019-47381-4 (PMC6659691; doi:10.1038/s41598-019-47381-4)
Supplement: Supplementary file 1 — Supplementary Dataset 1 [file 41598_2019_47381_MOESM1_ESM.pdf]

Computational Models Accurately Predict Multi-Cell Biomarker Profiles in Inflammation and Cancer

Carol L Fischer[1], Amber M Bates[2], Emily Lanzel[3], Janet M Guthmiller[4], Georgia K Johnson[5], Neeraj Kumar Singh[6], Ansu Kumar[6], Robinson Vidva[6], Taher Abbasi[6], Shireen Vali[6], Xian Jin Xie[7], Erliang Zeng[7], & Kim A Brogden[5,8*]

[1]Department of Biology, Waldorf University, Forest City, IA 50436 USA. [2]Department of Human Oncology, University of Wisconsin School of Medicine and Public Health, University of Wisconsin-Madison, Madison, WI 53705 USA. [3]Department of Oral Pathology, Radiology and Medicine, College of Dentistry, University of Iowa, Iowa City, IA 52242 USA. [4]College of Dentistry, University of Nebraska Medical Center, Lincoln, NE 68583 USA. [5]Department of Periodontics, College of Dentistry, the University of Iowa, Iowa City, IA 52242 USA. [6]Cellworks Group Inc., San Jose, CA 95110 USA and Cellworks Research India Pvt. Ltd, Bangalore, India (Wholly owned subsidiary of Cellworks Group Inc.). [7]Division of Biostatistics and Computational Biology, College of Dentistry, The University of Iowa, Iowa City, IA 52242 USA. [8]Iowa Institute for Oral Health Research, College of Dentistry, the University of Iowa, Iowa City, IA 52242, USA. Correspondence and requests for materials should be addressed to K.A.B. (email: *kim-brogden@uiowa.edu*)

Subject Areas: Computational models, Inflammation, Cancer, Chemokines, Cytokines

Supplementary Fig. S1. Cell-type specific computational models created for a variety of single myeloid, lymphoid, epithelial, miscellaneous, and cancer cells. In this study, we used single cell computational models for keratinocytes, dendritic cells, helper T lymphocytes, and multiple myeloma cells to prepare multi-cell computational models.

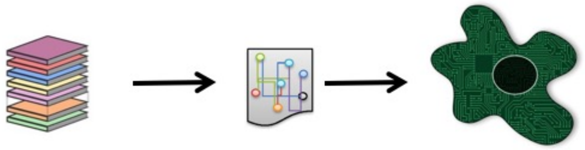

**Myeloid cells**	**Lymphoid cells**	**Epithelial cells**	**Miscellaneous cells**	**Cancer cells**
$CD34^+$ bone marrow cells<br>Stromal cells[1]<br>Monocytes<br>Dendritic cells[2,3]	Helper T lymphocytes[4]	Epithelial cells[1]<br>Keratinocytes[4]	Dermal fibroblasts[5]<br>Myometrial cells[6]<br>Dopaminergic cells[7]	Cancer cells[1]<br>Hepatocellular carcinoma cells[8]<br>Gastric cancer cells[9]<br>Glioblastoma[10]<br>Multiple myeloma[11,12]<br>HNSCC[12,13]<br>Cells for solid tumors<br>Cells for heme cancers

References

1 Vali, S., Pallavi, R., Kapoor, S. & Tatu, U. Virtual prototyping study shows increased ATPase activity of Hsp90 to be the key determinant of cancer phenotype. *Syst. Synth. Biol.* **4**, 25-33, https://doi.org/10.1007/s11693-009-9046-3 (2010).

2 Harvey, L. E. *et al.* Defensin DEFB103 bidirectionally regulates chemokine and cytokine responses to a pro-inflammatory stimulus. *Sci. Rep.* **3**, 1232, https://doi.org/10.1038/srep01232 (2013).

3 Borgwardt, D. S. *et al.* Histatin 5 binds to *Porphyromonas gingivalis* hemagglutinin B (HagB) and alters HagB-induced chemokine responses. *Sci. Rep.* **4**, 3904, https://doi.org/10.1038/srep03904 (2014).

4 Fischer, C. L. *et al.* A Predictive Model of an Oral Inflammatory Response. *J. Dent. Res.* **93 (A)**, Abstract 1369 (2014).

5 Almine, J. F. *et al.* Elastin sequences trigger transient proinflammatory responses by human dermal fibroblasts. *FASEB J.* **27**, 3455-3465, https://doi.org/10.1096/fj.13-231787 (2013).

6 Equils, O. *et al.* A computer simulation of progesterone and Cox2 inhibitor treatment for preterm labor. *PLoS ONE* **5**, e8502, https://doi.org/10.1371/journal.pone.0008502 (2010).

7 Sultana, Z. *et al.* Dynamic modeling of alpha-synuclein aggregation in dopaminergic neuronal system indicates points of neuroprotective intervention: experimental validation with implications for Parkinson's therapy. *Neuroscience* **199**, 303-317, https://doi.org/10.1016/j.neuroscience.2011.10.018 (2011).

8 Rajendran, P. *et al.* Suppression of signal transducer and activator of transcription 3 activation by butein inhibits growth of human hepatocellular carcinoma in vivo. *Clin. Cancer Res.* **17**, 1425-1439, https://doi.org/10.1158/1078-0432.CCR-10-1123 (2011).

9 Ramachandran, L. *et al.* Isorhamnetin inhibits proliferation and invasion and induces apoptosis through the modulation of peroxisome proliferator-activated receptor gamma activation pathway in gastric cancer. *J. Biol. Chem.* **287**, 38028-38040, https://doi.org/10.1074/jbc.M112.388702 (2012).

10 Pingle, S. C. *et al.* In silico modeling predicts drug sensitivity of patient-derived cancer cells. *J. Transl. Med.* **12**, 128, https://doi.org/10.1186/1479-5876-12-128 (2014).

11 Doudican, N. A. *et al.* Personalization of cancer treatment using predictive simulation. *J. Transl. Med.* **13**, 43, https://doi.org/10.1186/s12967-015-0399-y (2015).

12 Lanzel, E. A. *et al.* Predicting PD-L1 expression on human cancer cells using next-generation sequencing information in computational simulation models. *Cancer Immunol. Immunother.* **65**, 1511-1522, https://doi.org/10.1007/s00262-016-1907-5 (2016).

13 Bates, A. M. *et al.* Cell genomics and immunosuppressive biomarker expression influence PD-L1 immunotherapy treatment responses in HNSCC - a computational study. *Oral Surg. Oral Med. Oral Pathol. Oral Radiol.* **124**, 157-164, https://doi.org/10.1016/j.oooo.2017.05.474 (2017).

Supplementary Fig S2. Cultures of primary oral gingival epithelial keratinocytes (GE KER), dendritic cells (DC), and helper T lymphocytes (HTL) were exposed to LPS. The CSF2 (GM-CSF), CCL3 (MIP1α), CSF2 (GM-CSF), CCL5 (RANTES), IL1α, IL6, IL8, TNFα, IL12(p40), and VEGF responses were determined. Cells had match rates among predicted and observed responses ranging from 80-90%.

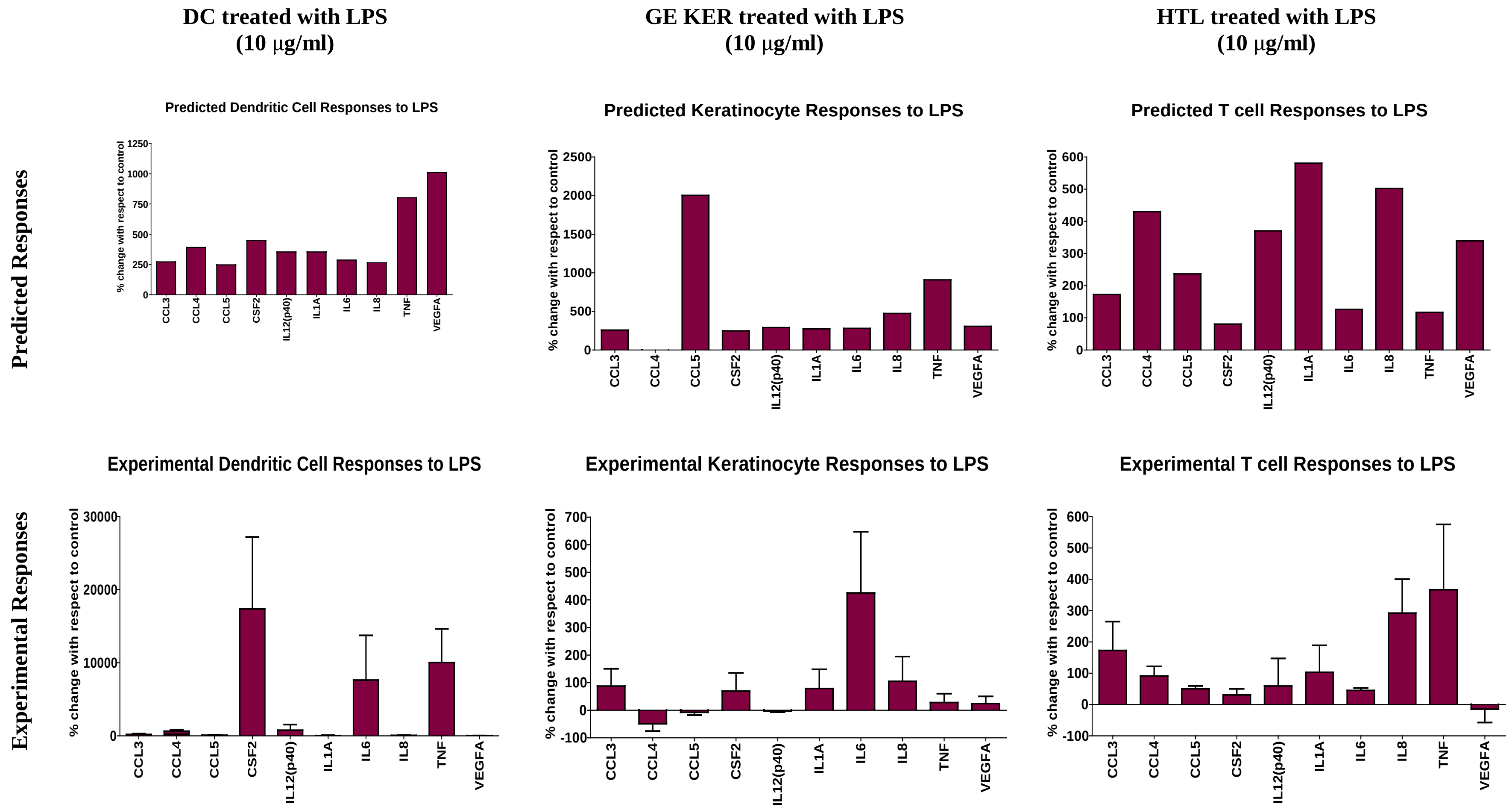

Supplementary Fig. S3. Single and multi-cell cultures of dendritic cells (DC) and multiple myeloma (MM) cell lines MM.1S and U266B1. Signals in multi-cell cultures influence the production of immunosuppressive biomarkers in the culture supernatants and on the cell surfaces. One-way fixed-effect ANOVA. Pairwise group comparisons were conducted using the method of Tukey's Honestly Significant Difference (HSD). Bars with the same letter(s) were not significantly different. (p>0.05; JMP, V10.0, SAS, Cary, NC).

a

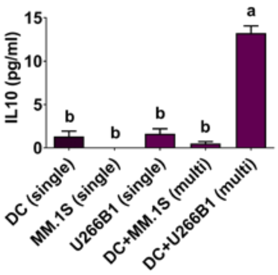

b

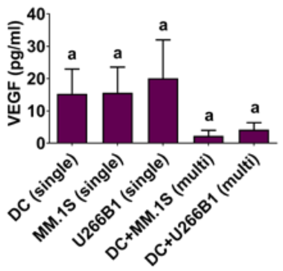

c

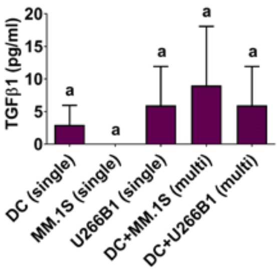

d

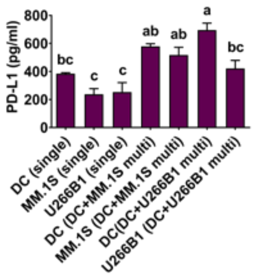

e

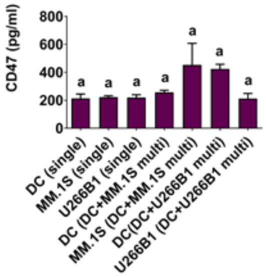

f

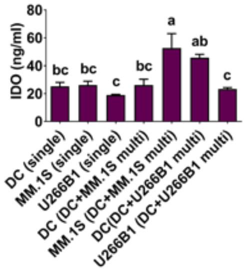

g

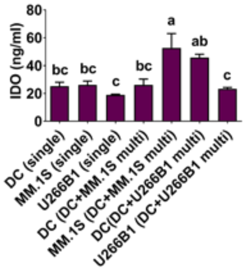

Supplementary Fig. S4. Multiple myeloma (MM) and dendritic cell (DC) multi-cell computational models of cancer. The effect of 23 MM lines (listed in the columns in the first row) on 19 dendritic cell biomarker readout responses (listed in the rows of the left-hand column) are shown. The colors in this dataset represent vast effects of MM cells on DC biomarker readouts. High (green), moderate (yellow), and low (red) expression levels were predicted and the specific numbers indicate the percent changes from control baseline, respectively.

Study	CONTROL VALUE	OPM2	RPMI8226	U266	LP1	OPM1	MM1S	ARH77	ARP1	SUDHL4	KMS11	MDSL	IM9	MDS92	L363	H929	MWCL1	MUTZ1	KMS12PE	JEKO1	RPCIWM1	SKM1	SKMM2	K562
CD80	7.74E-06	-72.0%	-52.9%	-69.4%	-75.3%	-54.2%	-19.3%	19.0%	-35.3%	-27.6%	30.4%	-43.4%	-25.9%	-27.6%	-50.9%	-27.1%	-36.8%	-42.7%	-39.0%	-47.3%	0.8%	-49.8%	-36.0%	-67.0%
CD86	2.71E-04	-74.9%	-54.7%	-72.3%	-79.6%	-55.7%	-16.3%	22.6%	-33.9%	-25.3%	33.6%	-44.9%	-23.9%	-26.1%	-51.7%	-24.5%	-35.8%	-41.9%	-38.2%	-49.1%	5.0%	-51.0%	-34.6%	-69.2%
IL12A	3.51E-04	-81.6%	-74.0%	-86.7%	-79.4%	-72.7%	-43.4%	-9.6%	-55.4%	-50.8%	0.0%	-69.9%	-51.6%	-54.9%	-67.5%	-46.6%	-57.4%	-59.1%	-59.3%	-73.4%	-16.9%	-71.4%	-55.8%	-74.7%
IL12B	3.93E-02	-82.4%	-60.9%	-85.5%	-82.5%	-60.3%	-12.5%	38.7%	-29.4%	-20.0%	54.8%	-50.6%	-22.7%	-27.9%	-52.4%	-20.3%	-32.1%	-40.1%	-35.4%	-58.7%	25.5%	-55.0%	-29.9%	-72.0%
IFNG	1.90E-03	-89.8%	-83.6%	-89.2%	-90.4%	-83.4%	-61.5%	-26.1%	-71.9%	-69.4%	-15.9%	-80.6%	-67.9%	-70.8%	-80.6%	-64.8%	-74.8%	-74.6%	-74.7%	-83.3%	-40.6%	-82.0%	-71.8%	-85.3%
IFNA1	2.13E-07	-43.8%	-34.9%	-50.9%	-40.7%	-35.5%	-12.9%	-4.8%	-24.4%	-20.9%	-3.6%	-24.3%	-16.2%	-14.0%	-32.7%	-15.7%	-25.0%	-24.8%	-26.8%	-27.8%	-9.5%	-33.8%	-25.4%	-34.7%
IFNB1	2.73E-05	-87.4%	-81.7%	-92.9%	-85.6%	-80.2%	-54.4%	-19.8%	-65.1%	-61.1%	-9.0%	-79.4%	-62.6%	-66.0%	-75.2%	-56.6%	-66.5%	-67.8%	-68.7%	-80.0%	-24.6%	-79.7%	-65.6%	-81.0%
IL2	8.14E-04	-95.9%	-94.5%	-96.4%	-95.3%	-94.3%	-79.1%	-39.6%	-87.4%	-85.9%	-26.0%	-93.0%	-84.6%	-86.9%	-92.7%	-81.8%	-89.5%	-89.0%	-89.4%	-94.6%	-58.0%	-93.8%	-87.3%	-93.8%
MHC-I	2.85E-09	-50.8%	15.2%	-52.6%	-29.3%	3.3%	322.2%	690.0%	118.3%	159.6%	782.4%	143.8%	214.2%	196.6%	31.5%	199.0%	116.2%	75.1%	93.1%	49.4%	489.8%	23.3%	117.8%	-29.9%
MHC-II	2.46E-08	-88.9%	-59.1%	-88.6%	-85.5%	-63.0%	133.4%	512.1%	0.8%	32.8%	654.9%	-20.8%	44.5%	22.9%	-43.9%	70.2%	10.0%	-17.4%	-16.5%	-31.2%	400.7%	-58.7%	-1.7%	-80.1%
IL3	1.30E-04	-34.1%	-1.1%	-21.2%	-41.9%	-4.0%	37.4%	64.0%	19.9%	31.2%	71.2%	14.0%	32.5%	32.5%	-0.6%	27.3%	20.4%	8.9%	16.1%	11.1%	50.7%	3.5%	18.6%	-29.4%
OSM	7.70E-06	-62.6%	-1.4%	-39.4%	-75.8%	-7.2%	83.6%	153.4%	43.1%	69.1%	174.2%	29.7%	71.9%	71.7%	-0.4%	59.1%	44.1%	18.9%	34.8%	24.0%	117.4%	8.0%	40.3%	-54.6%
TNFSF13B	8.50E-03	241.1%	227.4%	255.3%	241.6%	225.6%	157.7%	73.4%	193.4%	175.9%	38.5%	246.1%	195.2%	213.1%	198.4%	153.2%	173.9%	187.2%	203.8%	180.9%	40.9%	241.5%	196.9%	236.2%
THBS1	1.10E-03	190.6%	130.7%	897.1%	151.3%	134.0%	46.4%	38.1%	61.8%	72.1%	41.8%	77.5%	49.0%	55.2%	112.9%	51.3%	105.1%	65.6%	69.0%	442.9%	59.5%	98.0%	55.4%	49.6%
CXCL12	1.04E-04	94.7%	90.0%	105.8%	135.8%	87.5%	64.5%	42.6%	76.5%	69.9%	36.2%	118.8%	81.9%	94.6%	73.6%	59.7%	69.0%	69.2%	80.9%	68.9%	27.7%	101.3%	77.4%	91.9%
CXCL11	3.98E-03	396.2%	269.5%	996.5%	337.9%	278.2%	64.5%	31.1%	115.3%	130.4%	36.5%	148.5%	73.5%	87.0%	236.6%	84.2%	208.7%	131.2%	133.7%	684.2%	94.6%	203.0%	101.6%	128.2%
FN1	1.05E-02	222.4%	146.4%	591.5%	185.5%	151.9%	27.5%	17.2%	55.3%	66.0%	23.6%	72.5%	30.9%	38.4%	127.1%	38.5%	112.3%	64.0%	65.7%	401.7%	53.4%	105.9%	46.7%	56.9%
IL10	1.06E-03	-10.8%	-12.0%	148.0%	-11.7%	-11.0%	2.3%	28.7%	-7.7%	1.4%	38.0%	-15.7%	-4.3%	-5.0%	-9.5%	-1.7%	2.8%	-12.5%	-10.0%	66.3%	25.3%	-17.0%	-10.2%	-37.1%
Cell Count	146.50	-83.2%	-77.0%	-87.4%	-80.5%	-76.4%	-49.1%	-14.6%	-60.9%	-58.0%	-7.7%	-72.0%	-55.5%	-57.7%	-72.5%	-53.4%	-64.4%	-65.2%	-64.5%	-77.2%	-32.1%	-74.3%	-61.2%	-77.8%

Supplementary Table S1. Examples of cells cultivated i) in single cell or heterotypic co-culture of cells in spheroids, organoids, and tumoriods or transwell co-cultures and ii) on scaffold-based systems to assess bio-matrices that contain structural proteins and growth factors important in tissue organization.

**Liquid-based systems**
Single cell or heterotypic co-culture of cells in spheroids, organoids, and tumoriods
• Hanging drop tumor spheroids of tumor cells, fibroblasts, and immune cells were treated with a novel immunocytokine (interleukin-2 variant; IgG-IL2v) and tumor- or fibroblast-targeted T cell bispecific antibody, which activated T cells, NK cells, and NKT cells whereas the combination of TCBs with IgG-IL2v molecules enhanced immune cell infiltration; activation; increased cytokine secretion[1]
• Hanging drop tumor spheroids of endothelial cells and melanoma cancer stem-like cells were used to assess the cellular cross-talk of the angiogenic microenvironment[2]
• Floating liquid marble spheroids of olfactory ensheathing cells formed extensive cell-to-cell connections with co-cultures of Schwann cells, and astrocytes[3]
• Gel-based' arrays of spheroids of multiple cell types had differing 3D morphologies and IHC biomarker expression profiles[4]
• Pellet culture method and Rotary Cell Culture System generated spheroids were analyzed via image analysis to correlate spheroid volume and shape with reproducibility of data results[5]
• Organoids of cells from three colorectal cancers and cells from adjacent normal intestinal crypts were assessed for mutational profiles and colorectal cancer cells experienced substantial increases in somatic mutation rate compared to normal colorectal cells with marked differences in responses to anticancer drugs between even closely related cells of the same tumor[6,7]
• Patient-derived tumor organoids were used in high-throughput drug screening assays and cells from two uterine malignancies and two colon cancers identified effective drugs and drug combinations that were subsequently validated using PDX models[8]
• Primary liver cancer organoids of hepatocellular carcinoma, cholangiocarcinoma, and combined tumors were amenable for biomarker identification and drug screening, the latter leading to the identification of ERK inhibitors[9]
Transwell co-cultures
• Transwell co-cultures of human pancreatic cancer cells and human pancreatic stellate cells had increased levels of TGFβ with enhanced levels of pSMAD2 and p21 signaling suggesting a synergistic effect of the stroma to epithelial TGFβ signaling[10]
• Transwell co-cultures of macrophages, fibroblasts, and myoblasts altered myoblast proliferation and migration[11]
• Airlifted transwell co-cultures of squamous cell carcinoma cells and fibroblasts formed a non-keratinizing, multilayer squamous epithelium. 5-FU, inhibited cell proliferation, decreased E-cadherin production, did not alter biofim growth, and increased microbial-induced pro-inflammatory cytokine production[12]
**Scaffold-based systems**
Single cell or heterotypic co-cultures of cells cultivated on 3D scaffolds
• PLGA scaffolds of MC3T3-E1 osteoprogenitor cells produced lower expression levels of alkaline phosphatase and osteocalcin, but elevated expression levels of VEGF[13]
• Collagen-elastin matrix with gingival fibroblasts and keratinocytes formed multi-layered oral mucosa-like structures applicable for studies in periodontal therapy[14]
• Bovine fibronectin matrix with colorectal or breast cancer cells and stromal cells in perfused microvessels formed vascularized microtumors, which can be regressed by drugs that target VEGFRs, PDGFR and Tie2[15]
• Peptide functionalized PEG hydrogels with dermal fibroblasts had melanoma cell influenced levels of cell migration, cluster growth, and invasion[16]
• Laminin-rich scaffolds of luminal MCF-7 cells or basal MDA-MB-231 cells had different morphologies, cell-cell interactions, and biomarker expression profiles in the presence of T_{REG} lymphocytes and NK cells[17]
• Air exposed methyl cellulose supported membranes with keratinocyte-bacterial, keratinocyte-to-keratinocyte, and keratinocyte-to-fibroblast mixtures formed stratified multi-layers with unique agonist-induced biomarker profiles[18,19]
• Porcine collagen, type I scaffolds with keratinocyte, fibroblast, and monocyte organotypic tissue produced cytokines in the presence of an 11-microbial species biofilm challenge[20]

References

1 Herter, S. *et al.* A novel three-dimensional heterotypic spheroid model for the assessment of the activity of cancer immunotherapy agents. *Cancer Immunol. Immunother.* **66**, 129-140, https://doi.org/10.1007/s00262-016-1927-1 (2017).

2 Klimkiewicz, K. *et al.* A 3D model of tumour angiogenic microenvironment to monitor hypoxia effects on cell interactions and cancer stem cell selection. *Cancer Lett.* **396**, 10-20, https://doi.org/10.1016/j.canlet.2017.03.006 (2017).

3 Vadivelu, R. K. *et al.* Generation of three-dimensional multiple spheroid model of olfactory ensheathing cells using floating liquid marbles. *Sci. Rep.* **5**, 15083, https://doi.org/10.1038/srep15083 (2015).

4 Ivanov, D. P. & Grabowska, A. M. Spheroid arrays for high-throughput single-cell analysis of spatial patterns and biomarker expression in 3D. *Sci. Rep.* **7**, 41160, https://doi.org/10.1038/srep41160 (2017).

5 Zanoni, M. *et al.* 3D tumor spheroid models for in vitro therapeutic screening: a systematic approach to enhance the biological relevance of data obtained. *Sci. Rep.* **6**, 19103, https://doi.org/10.1038/srep19103 (2016).

6 Sato, T. *et al.* Long-term expansion of epithelial organoids from human colon, adenoma, adenocarcinoma, and Barrett's epithelium. *Gastroenterology* **141**, 1762-1772, https://doi.org/10.1053/j.gastro.2011.07.050 (2011).

7 Roerink, S. F. *et al.* Intra-tumour diversification in colorectal cancer at the single-cell level. *Nature* **556**, 457-462, https://doi.org/10.1038/s41586-018-0024-3 (2018).

8 Pauli, C. *et al.* Personalized In Vitro and In Vivo Cancer Models to Guide Precision Medicine. *Cancer Discov.* **7**, 462-477, https://doi.org/10.1158/2159-8290.CD-16-1154 (2017).

9 Broutier, L. *et al.* Human primary liver cancer-derived organoid cultures for disease modeling and drug screening. *Nat. Med.*, https://doi.org/10.1038/nm.4438 (2017).

10 Principe, D. R. *et al.* TGFbeta Signaling in the Pancreatic Tumor Microenvironment Promotes Fibrosis and Immune Evasion to Facilitate Tumorigenesis. *Cancer Res.* **76**, 2525-2539, https://doi.org/10.1158/0008-5472.CAN-15-1293 (2016).

11 Venter, C. & Niesler, C. A triple co-culture method to investigate the effect of macrophages and fibroblasts on myoblast proliferation and migration. *Biotechniques* **64**, 52-58, https://doi.org/10.2144/btn-2017-0100 (2018).

12 Sobue, T. *et al.* Chemotherapy-induced oral mucositis and associated infections in a novel organotypic model. *Mol. Oral Microbiol.* **33**, 212-223, https://doi.org/10.1111/omi.12214 (2018).

13 Jarrahy, R. *et al.* Osteogenic differentiation is inhibited and angiogenic expression is enhanced in MC3T3-E1 cells cultured on three-dimensional scaffolds. *Am. J. Physiol. Cell Physiol.* **289**, C408-414, https://doi.org/10.1152/ajpcell.00196.2004 (2005).

14 Golinski, P. A., Groger, S., Herrmann, J. M., Bernd, A. & Meyle, J. Oral mucosa model based on a collagen-elastin matrix. *J. Periodontal Res.* **46**, 704-711, https://doi.org/10.1111/j.1600-0765.2011.01393.x (2011).

15 Sobrino, A. *et al.* 3D microtumors in vitro supported by perfused vascular networks. *Sci. Rep.* **6**, 31589, https://doi.org/10.1038/srep31589 (2016).

16 Singh, S. P. *et al.* A synthetic modular approach for modeling the role of the 3D microenvironment in tumor progression. *Sci. Rep.* **5**, 17814, https://doi.org/10.1038/srep17814 (2015).

17 Augustine, T. N., Dix-Peek, T., Duarte, R. & Candy, G. P. Establishment of a heterotypic 3D culture system to evaluate the interaction of TREG lymphocytes and NK cells with breast cancer. *J. Immunol. Methods* **426**, 1-13, https://doi.org/10.1016/j.jim.2015.07.003 (2015).

18 Gursoy, U. K. *et al.* Construction and characterization of a multilayered gingival keratinocyte culture model: the TURK-U model. *Cytotechnology* **68**, 2345-2354, https://doi.org/10.1007/s10616-016-0029-4 (2016).

19 Bedran, T. B., Mayer, M. P., Spolidorio, D. P. & Grenier, D. Synergistic anti-inflammatory activity of the antimicrobial peptides human beta-defensin-3 (hBD-3) and cathelicidin (LL-37) in a three-dimensional co-culture model of gingival epithelial cells and fibroblasts. *PLoS ONE* **9**, e106766, https://doi.org/10.1371/journal.pone.0106766 (2014).

20 Bao, K., Papadimitropoulos, A., Akgul, B., Belibasakis, G. N. & Bostanci, N. Establishment of an oral infection model resembling the periodontal pocket in a perfusion bioreactor system. *Virulence* **6**, 265-273, https://doi.org/10.4161/21505594.2014.978721 (2015).

<table>
<tr><td colspan="2">Supplementary Table S2. Dendritic cell computational model[1,2] was extensively validated against published laboratory studies.</td></tr>
<tr><td colspan="2">1. Effect of IL6 over-expression in dendritic cells cultured with IL4 and CSF2 and later stimulated with LPS[3].</td></tr>
<tr><td>EXPERIMENTAL</td><td>PREDICTIVE</td></tr>
<tr><td>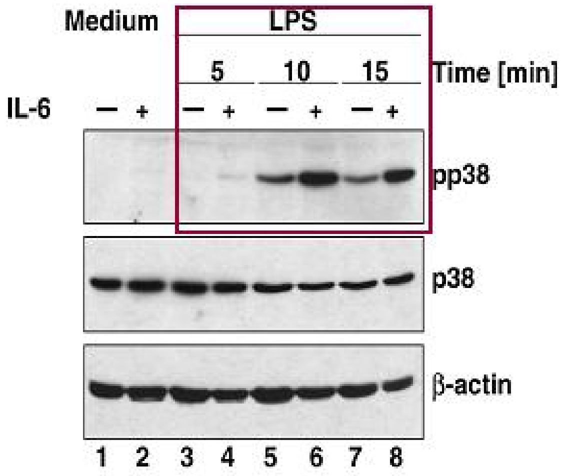
</td><td>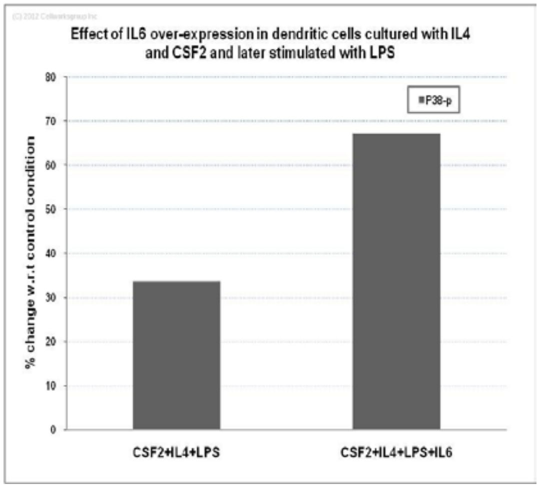
</td></tr>
<tr><td>Fig. 5. p38 MAPK activation is not suppressed in IL-6-DC: Human blood monocytes were cultured with GM-CSF (100 ng/ml) and IL-4 (5 ng/ml) in the absence (Lanes 1, 3, 5, and 7) or presence of IL-6 (100 ng/ml, Lanes 2, 4, 6, and 8). On day 6 the cells were stimulated with medium (Lanes 1 and 2) or LPS (250 ng/ml, Lanes 3–8) for the indicated time intervals. Whole cell extracts were prepared and analyzed by Western blot using phosphorylation-specific (top panel) or non-phosphorylation-specific Abs directed against the p38 MAPK (middle panel). Equal loading was controlled using a □-actin specific mAb (bottom panel).</td><td>The levels of phosphorylated P38 in the virtual cell system with GM-CSF (CSF2) and IL4 over-expressed by 5 folds, IL6 over-expressed by 10 folds and stimulated with 0.00001 uM LPS are compared with those in the virtual cell system with GM-CSF (CSF2) and IL4 over-expressed by 5 folds and stimulated with 0.00001 uM LPS alone.</td></tr>
<tr><td>EXPERIMENTAL</td><td>PREDICTIVE</td></tr>
</table>

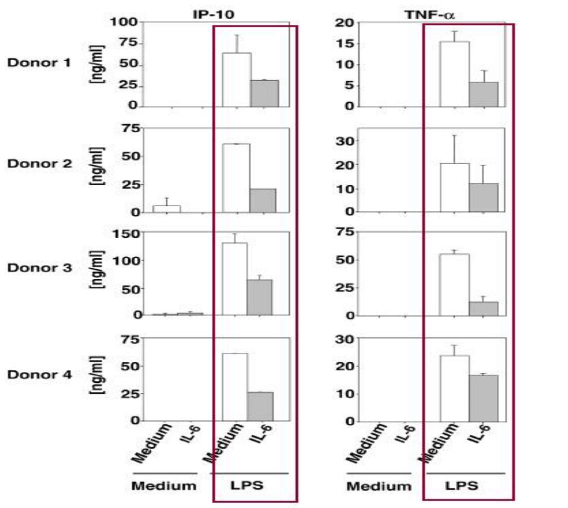

Fig. 6. IL-6 reduces LPS-induced IP-10 and TNF-α production: Human blood monocytes from four different donors were cultured with GM-CSF (100 ng/ml) and IL-4 (5 ng/ml) either in the absence or presence of IL-6 (100 ng/ml). On day 6, they were stimulated with medium or LPS (250 ng/ml). Twenty-four hours after LPS stimulation supernatants were harvested and analysed for IP-10 and TNF-α levels (per 10^6 cells) by ELISA.

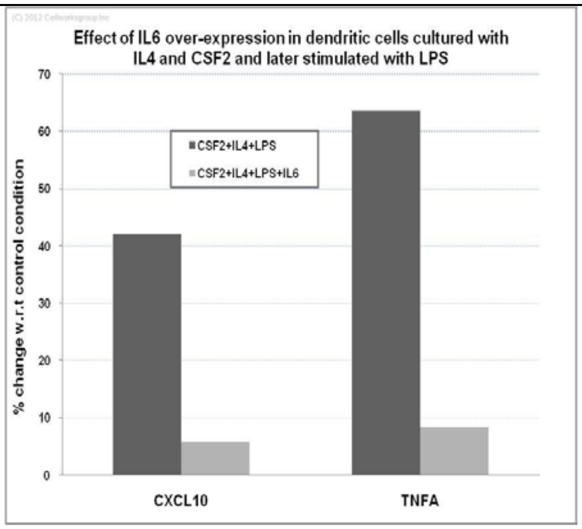

TNF-α and IP-10 (CXCL10) expression levels in the virtual cell system with GM-CSF (CSF2) and IL4 over-expressed by 5 folds, IL6 over-expressed by 10 folds and stimulated with 0.00001 uM LPS are compared with those in the virtual cell system with GM-CSF (CSF2) and IL4 over-expressed by 5 folds and stimulated with 0.00001 uM LPS alone.

Inference: IL6 inhibits LPS mediated TNF-α and IP-10 expression but increases LPS induced p38 phosphorylation.

2. Effect of PKC inhibitor on LPS stimulated dendritic cells[4].

EXPERIMENTAL	PREDICTIVE

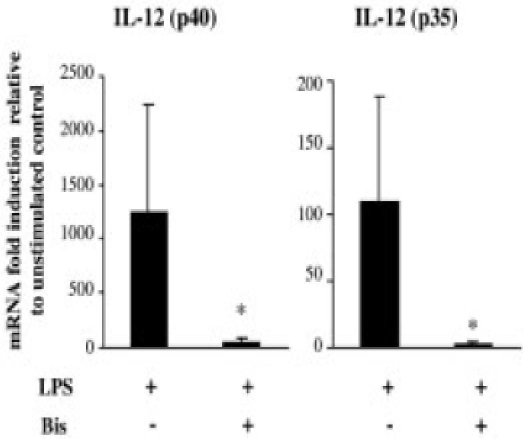

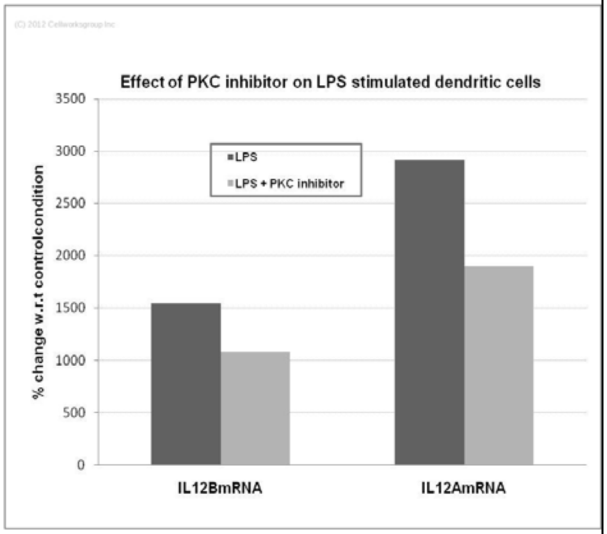

**Fig. 2B. Bis (Pan-PKC inhibitor) inhibits LPS-induced IL-12 synthesis.** DC (1×106/ml) were incubated with graded concentrations of Bis and subsequently stimulated for 6 h with LPS. IL-12 (p40) and IL-12 (p35) mRNA levels were quantified by real-time RT-PCR. IL-12 (p40) and IL-12 (p35) mRNA levels were normalized against g -Actin mRNA. Results are shown as mRNA fold induction mean ± SEM of six independent experiments on different donors.	IL12A (IL12-p35) and IL12 B (IL12-p40) mRNA levels in the virtual cell system stimulated with 0.0001 uM LPS and treated with PKC inhibitor (inhibiting the activity of different isoforms of PKC by around 75%) are compared with those in the virtual cell system stimulated with 0.0001 uM LPS alone.

Inference: Pan-PKC inhibitor decreases LPS mediated IL12A and IL12B mRNA levels in dendritic cells.

3. Effect of STAT3 knock out in CSF2 differentiated, LPS stimulated dendritic cells[5].

EXPERIMENTAL

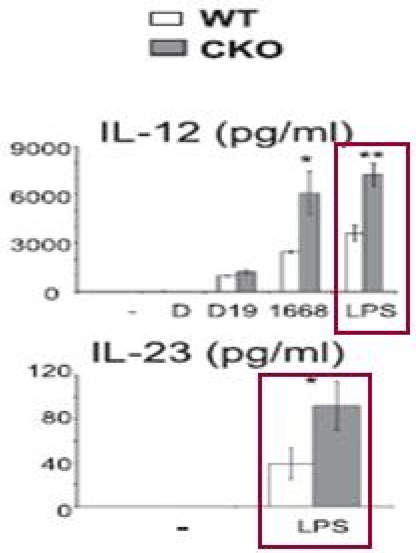

PREDICTIVE

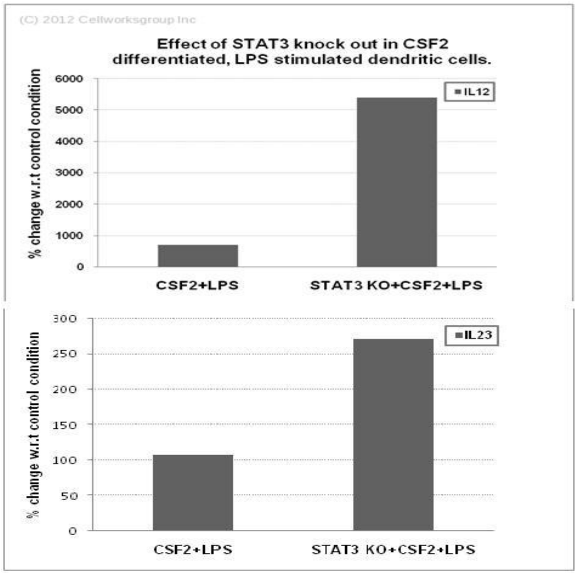

**Fig. 4. Functional characterization of Stat3 CKO DCs.** Stimulated CKO DCs exhibit enhanced capacity to secrete cytokines in vitro. WT and CKO BMDCs (day 6 GM-CSF) were evaluated for cytokine expression after stimulation with TLR agonists, including D (negative control CpG; 1 mM), D19 (A/D-type CpG; 1 mM), 1668 (K-type CpG; 1 mM), and LPS (1000 ng/ml). Twelve-hour supernatants were evaluated for cytokine production by ELISA (IL-23) or bead array (IL-12).	IL12 and IL23 expression levels in the virtual cell system with 100% knock-out of STAT3, 10 fold over-expression of CSF2, stimulated with 0.00001 uM LPS are compared with those in the virtual cell system with 10 fold over-expression of CSF2, stimulated with 0.00001 uM LPS alone.

Inference: STAT3 knock out in CSF2 differentiated, LPS stimulated dendritic cells leads to an increase in the expression levels of IL12 and IL23.

4. Effect of MSR1 knock out in CSF2 differentiated, LPS stimulated dendritic cells[6].

EXPERIMENTAL	PREDICTIVE
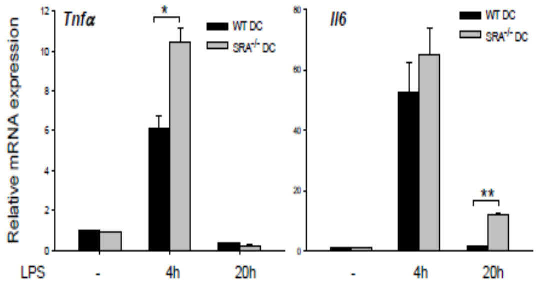	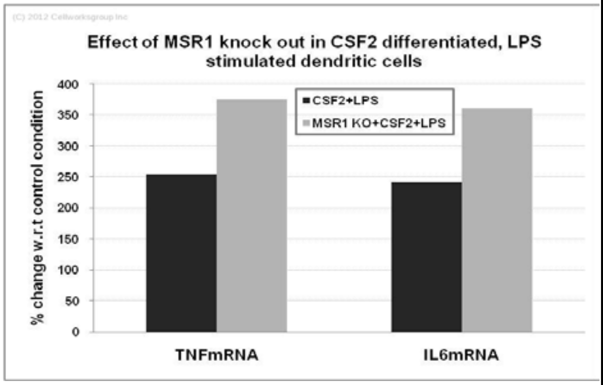
**Fig. 1** BMDCs were stimulated with 100ng/ml LPS and mRNA levels of inflammatory genes (*Tnfα and Il6*) were assessed using qRT-PCR. The results were presented as folds induction of treated over untreated WT samples.	mRNA levels of TNF-α and IL6 in the virtual cell system with 100% knock-out of MSR1, 10 fold over-expression of CSF2, stimulated with 0.0001 uM LPS are compared with those in the virtual cell system with 10 fold over-expression of CSF2, stimulated with 0.0001 uM LPS alone.

Inference: MSR1 knock out in CSF2 differentiated, LPS stimulated dendritic cells leads to an increase in the mRNA levels of IL6 and TNF

5. Effect of dsRNA (poly (I:C)) stimulation on CSF2 and IL4 differentiated dendritic cells[7].

EXPERIMENTAL	PREDICTIVE
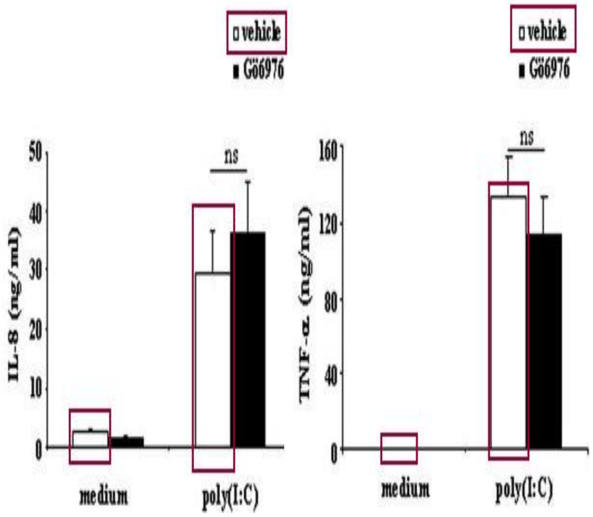	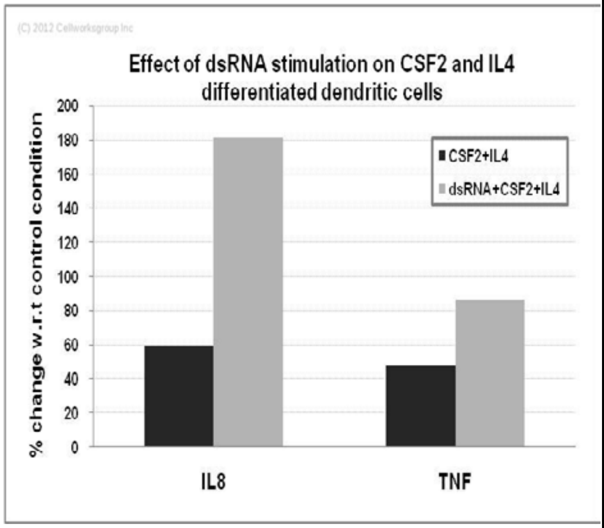

**Fig 1.** Immature DCs (CSF2 and IL4 differentiated) were incubated in vehicle (Me2SO) or the indicated concentrations of Go¨6976 for 2 h and then activated by poly(I:C) (10µg/ml) or left unstimulated. after 20 h, IL-8 (*left*) and tumor necrosis factor-α (*right*) levels in culture supernatants were analyzed by ELISA	IL8 and TNF-α expression levels in the virtual cell system with GM-CSF (CSF2) and IL4 over-expressed by 5 folds and stimulated with 0.00002 uM poly (I:C) are compared with those in the virtual cell system with GM-CSF (CSF2) and IL4 over-expressed by 5 folds.

Inference: dsRNA stimulation on CSF2 and IL4 differentiated dendritic cells leads to an increase in the levels of IL8 and TNFA

6. Effect of TLR4 knock out in CSF2 differentiated, HAGB stimulated dendritic cells[8].

EXPERIMENTAL	PREDICTIVE

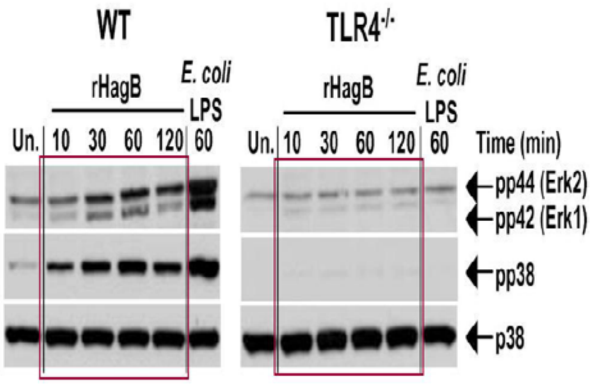

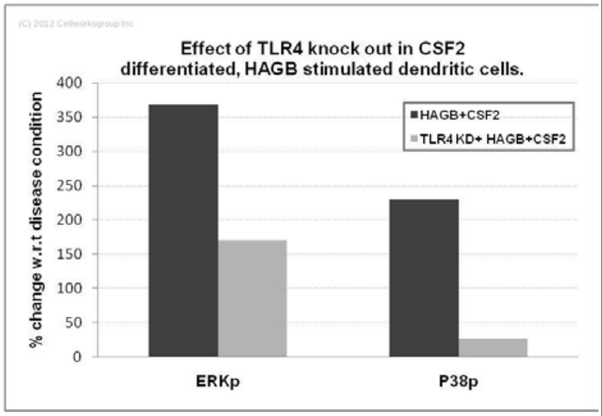

**Fig 6.** Phosphorylation of p38, ERK1/2 by rHagB stimulated DC is dependent on TLR4. DC from WT, and TLR4−/− mice were stimulated with 40 µg/ml rHagB for 10, 30, 60 or 120 min. Following stimulation, cells were lysed and whole cell lysates were assessed for ERK1/2 and p38 and by Western blot. Total p38 was used as loading controls. Unstimulated DC (far left lanes) or DC stimulated with 100 ng/ml *E. coli* K12 LPS for 60 min (far right lanes) were used as controls.	Levels of phosphorylated ERK and P38 in the virtual cell system with 100% knock-out of TLR4, 10 fold over-expression of CSF2 stimulated with 0.002 uM HAGB are compared with those in the virtual cell system with 10 fold over-expression of CSF2, stimulated with 0.002 uM HAGB alone.

Inference: TLR4 knock out in CSF2 differentiated, HAGB stimulated dendritic cells leads to a decrease in the levels of phosphorylated ERK and P38

7. Effect of PGF over-expression on CSF2 and IL4 differentiated, LPS stimulated dendritic cells[9].

EXPERIMENTAL	PREDICTIVE

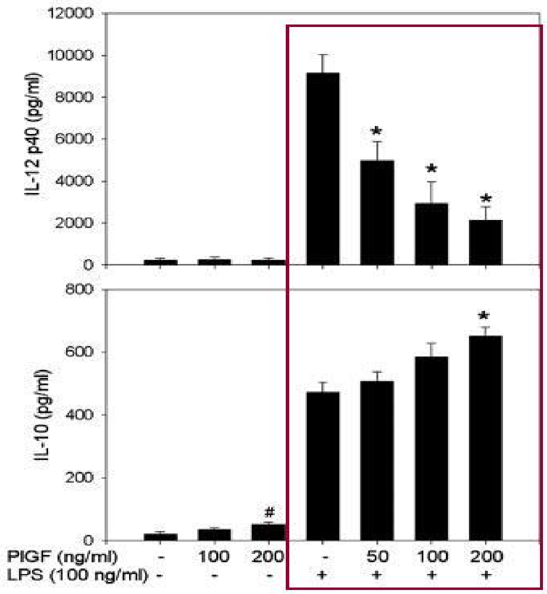

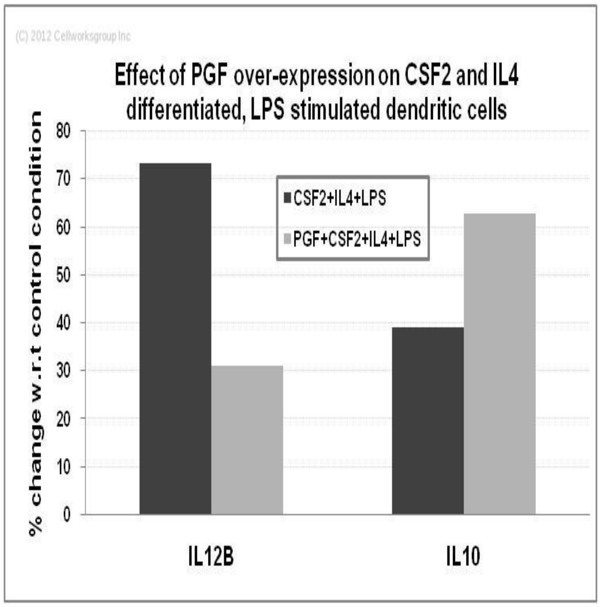

Fig. 1. Modulation of cytokine secretion of DC by PlGF. CD14+ monocytes were cultured with GM-CSF and IL-4 for 5 days to differentiate them into iDCs in absence of PlGF (referred as classical-DC). Classical-DC was treated with medium, various concentrations of PlGF, LPS (100 ng/ml) alone, or LPS plus various concentrations of PlGF for 48 h. At the end of the incubation time, IL-12 p40 and IL-10 production was subsequently analyzed by ELISA.

IL12B and IL10 expression levels in the virtual cell system with GM-CSF (CSF2) and IL4 over-expressed by 5 folds, PGF over-expressed by 100 folds and stimulated with 0.00001 uM LPS are compared with those in the virtual cell system with GM-CSF (CSF2) and IL4 over-expressed by 5 folds and stimulated with 0.00001 uM LPS alone.

Inference: PGF over-expression on CSF2 and IL4 differentiated, LPS stimulated dendritic cells leads to an decrease in the levels of IL12B and increase in the levels of IL10

8. Effect of mTOR inhibitor on LPS stimulated dendritic cells[10].

**EXPERIMENTAL**	**PREDICTIVE**

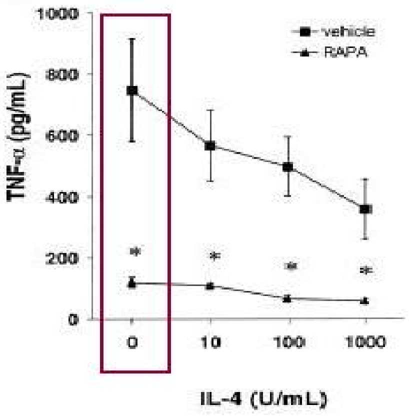

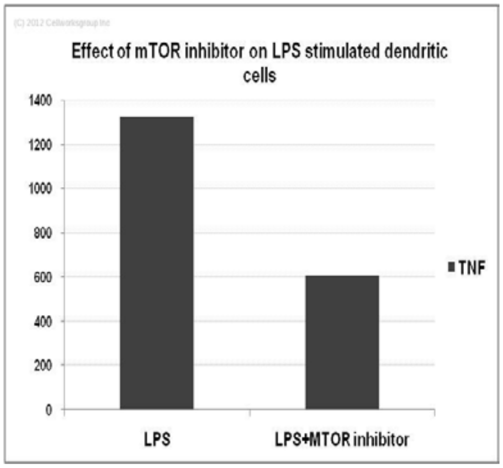

Fig. 6. Animals were treated with RAPA(OE) or vehicle (plus Flt3L, 10 days; f). Splenic DCs were purified by density gradient centrifugation and immunomagnetic bead sorting and stimulated with LPS for 24 hours. TNF-α production by DCs from mice given RAPA for 10 days versus drug vehicle-injected controls (3 animals/group) is given.

TNF-α expression levels in the virtual cell system stimulated with 0.0001 uM LPS and treated with mTOR inhibitor (giving approximately 75-80% on-target inhibition) are compared with those in the virtual cell system stimulated with 0.0001 uM LPS alone.

Inference: Introduction of mTOR inhibitor in LPS stimulated dendritic cells leads to a decrease in TNF-α levels.

9. Effect of SYK knock out in CSF2 differentiated, beta-glucan stimulated dendritic cells[11].

**EXPERIMENTAL**	**PREDICTIVE**

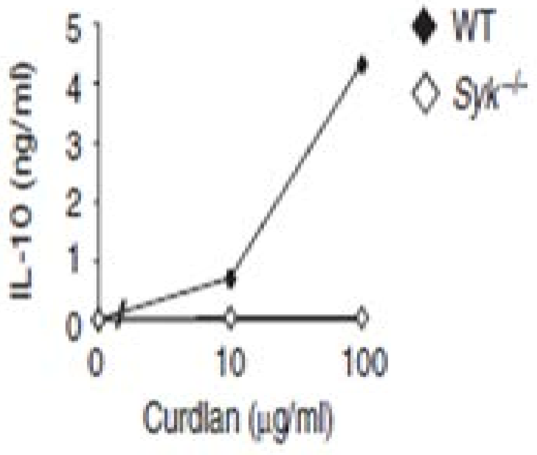

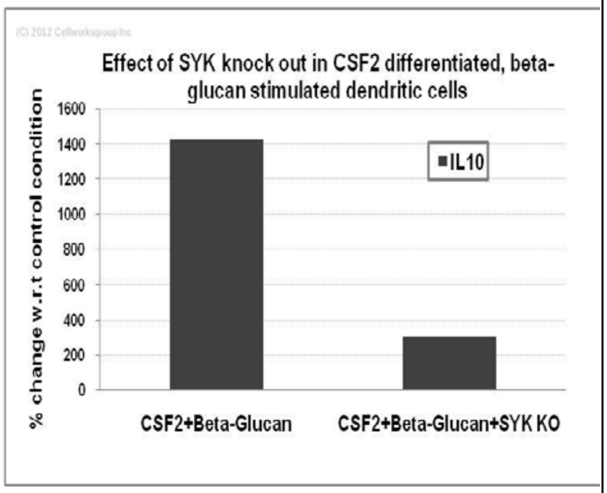

Fig. 1d. Production of IL-10 in response to curdlan depends on Syk. ELISA of IL-10 by BMDCs (cultured in GM-CSF media) of various genotypes after stimulation with curdlan (beta-glucan). C57BL/6 wild-type or Syk–/– BMDCs are stimulated with various amounts of curdlan (horizontal axis). Data are the mean ± s.d. of triplicate stimulations and are representative of two to six independent experiments.

IL10 expression levels in the virtual cell system with 100% knock-out of SYK, 10 fold over-expression of CSF2, stimulated with 0.00274 uM Beta-glucan are compared with those in the virtual cell system with 10 fold over-expression of CSF2, stimulated with 0.00274 uM Beta-glucan alone.

Inference: SYK knock out in CSF2 differentiated, beta-glucan stimulated dendritic cells leads to a decrease in the levels of IL10.

10. Effect of IFNG over-expression on CSF2 and IL4 differentiated, CD40LG stimulated dendritic cells[12].

EXPERIMENTAL	PREDICTIVE

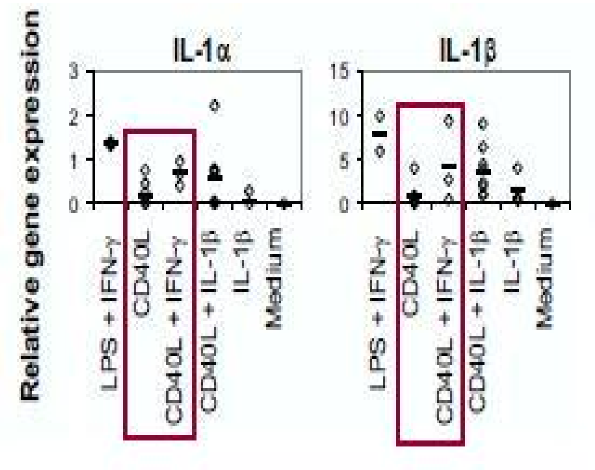

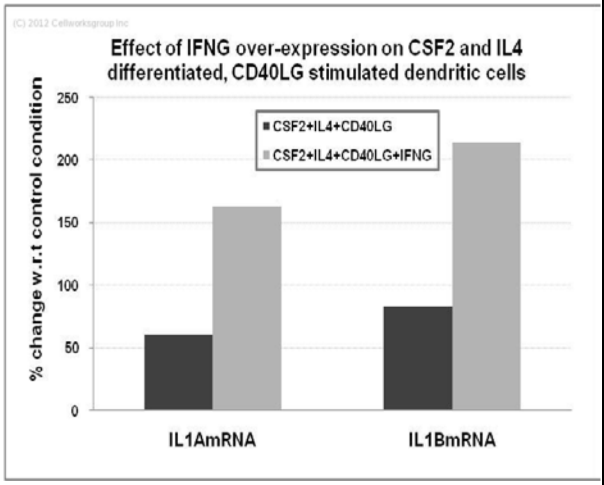

Fig 1. Comparison of different activation signals on gene expression in DC. Normalized gene expression levels from 2–7 independent experiments with averages indicated by a horizontal bar. DC were cultured with LPS+IFN-γ (n = 2); CD40L+IFN-γ (n = 3); CD40L+IL-1β (n = 7); CD40L (n = 7); IL-1β (n = 4); medium n = 4 for 8 hours and total RNA was extracted and hybridized to biotin-labeled RNA probes. After RNase treatment, digested probes were separated on a 5% acrylamide/8 M urea gel, blotted to a positively charged membrane for detection with CDP-Star substrate solution, and exposed to film or directly to the Kodak Image Station.

mRNA levels of IL1A and IL1B in the virtual cell system with GM-CSF (CSF2) and IL4 over-expressed by 5 folds, CD40LG over-expressed by 10 folds and IFNG over-expressed by 100 folds are compared with those in the virtual cell system with GM-CSF (CSF2) and IL4 over-expressed by 5 folds and CD40LG over-expressed by 10 folds.

Inference: IFNG over-expression on CSF2 and IL4 differentiated, CD40LG stimulated dendritic cells leads to an increase in the mRNA levels of IL1A and IL1B

11. Effect of activin receptor inhibitor on CSF2 and IL4 differentiated, activin stimulated dendritic cells[13].

EXPERIMENTAL	PREDICTIVE

EXPERIMENTAL

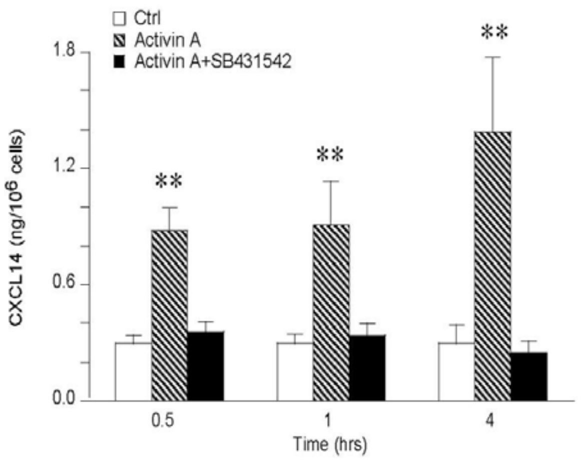

Fig. 4. Activin A up-regulates CXCL14 in DCs. Kinetics of CXCL14 release by 100 ng/mL activin-A–stimulated DCs (differentiated using GM-CSF and IL4). SB431542, an ALK4 (activin receptor) inhibitor, was used at the concentration of 5 μM.

PREDICTIVE

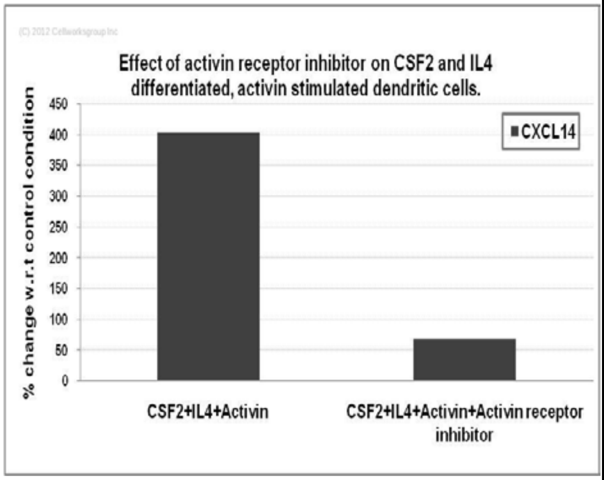

CXCL14 expression levels in the virtual cell system with GM-CSF (CSF2) and IL4 over-expressed by 5 folds, activin A over-expressed by 100 folds and treated with activin receptor (inhibiting the activity of the activin receptor by around 75-80%) are compared with those in the virtual cell system with GM-CSF (CSF2) and IL4 over-expressed by 5 folds and activin A over-expressed by 100 folds.

Inference: Introduction of activin receptor inhibitor on CSF2 and IL4 differentiated, activin stimulated dendritic cells leads to a decrease in the levels of CXCL14.

12. Effect of MYD88 and TICAM1 knock out in CSF2 differentiated, CpG-DNA stimulated dendritic cells[11].

EXPERIMENTAL

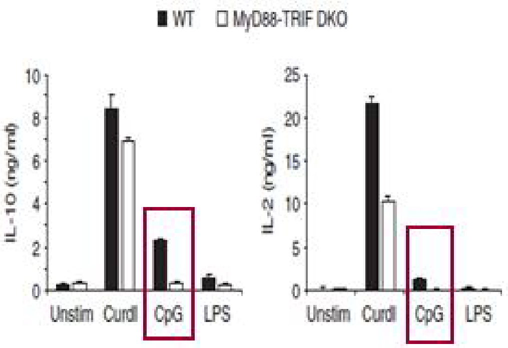

PREDICTIVE

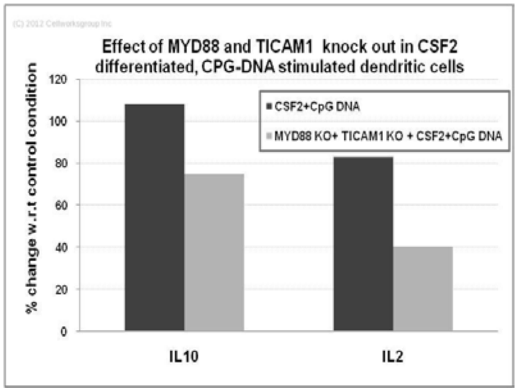

<table>
<tr><td>ELISA of IL-10 and IL-2 production by BMDCs (cultured in GM-CSF media) of various genotypes after stimulation with CpG. C57BL/6 wild-type or MyD88-TRIF–double-knockout (MyD88-TRIF DKO) BMDCs left unstimulated or stimulated with curdlan (Curdl), CpG or lipopolysaccharide (LPS). Data are the mean ± s.d. of triplicate stimulations and are representative of two to six independent experiments.</td><td>IL10 and IL2 expression levels in the virtual cell system with 100 % knock-out of MYD88 & TICAM (TRIF), with GM-CSF (CSF2) over-expressed by 10 folds and stimulated with 0.002 uM CpGDNA are compared with those in the virtual cell system with GM-CSF (CSF2) over-expressed by 10 folds and stimulated with 0.002 uM CpGDNA.</td></tr>
<tr><td colspan="2">Inference: MYD88 and TICAM1 knock out in CSF2 differentiated, CpG-DNA stimulated dendritic cells leads to a decrease in the levels of IL2 and IL10.</td></tr>
<tr><td colspan="2">13. Effect of Fractalkine stimulation on CSF2 and IL4 differentiated dendritic cells[14].</td></tr>
<tr><td>EXPERIMENTAL</td><td>PREDICTIVE</td></tr>
<tr><td>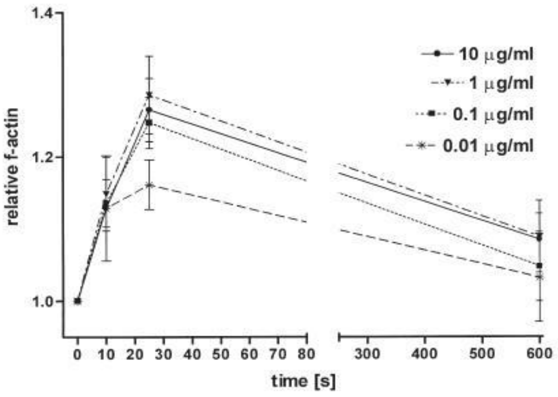
</td><td>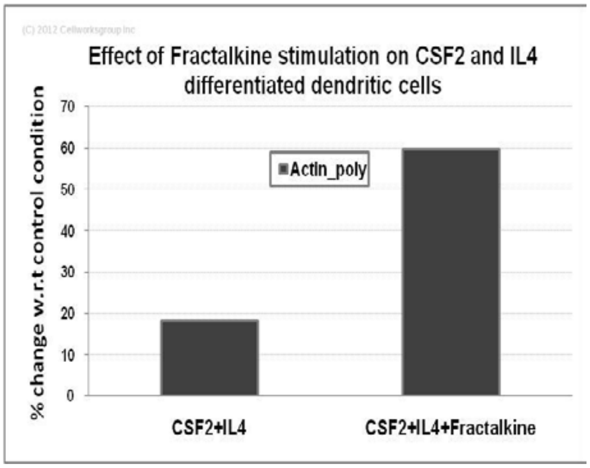
</td></tr>
<tr><td>Fig. 2. Fractalkine and actin polymerization of immature dendritic cells (GM-CSF and IL4 differentiated). Cells were stimulated with different concentrations of fractalkine. The relative f-actin content was determined at the indicated time points by flow cytometry. Data are means ± SEM (n=5 different donors).</td><td>f-Actin levels in the virtual cell system with GM-CSF (CSF2) and IL4 over-expressed by 5 folds and fractalkine over-expressed by 100 folds are compared with those in the virtual cell system with GM-CSF (CSF2) and IL4 over-expressed by 5 folds.</td></tr>
<tr><td colspan="2">Inference: Fractalkine stimulation on CSF2 and IL4 differentiated dendritic cells leads to an increase in the f-actin levels.</td></tr>
<tr><td colspan="2">14. Effect of p38-MAPK inhibitor on CSF2 and IL4 differentiated, CD40LG stimulated dendritic cells[15].</td></tr>
<tr><td>EXPERIMENTAL</td><td>PREDICTIVE</td></tr>
<tr><td></td><td></td></tr>
</table>

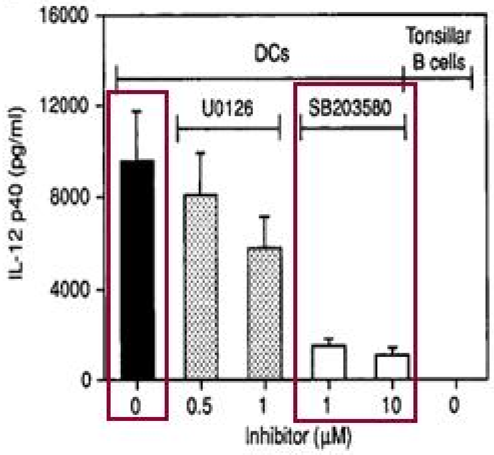

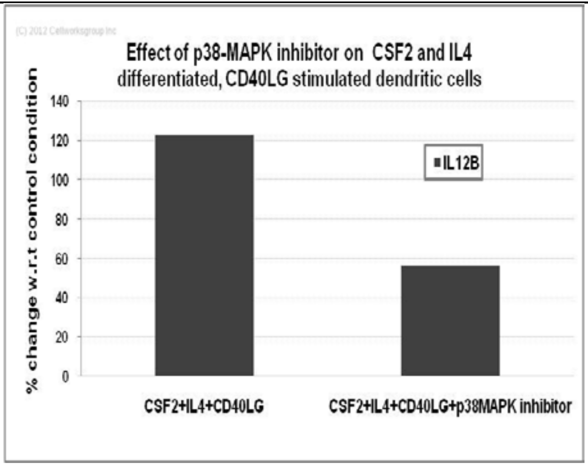

Fig. 4. CD40-induced IL-12 p40 protein secretion in DCs is inhibited by SB203580 (p38-MAPK inhibitor) in a dose-dependent manner, as measured by ELISA after 6 h of CD40 ligation. CD40-induced IL-12 p40 protein secretion in DCs is partially inhibited by U0126 (dotted bars) in a dose-dependent manner and compared with the stronger inhibition by SB203580 (open bars), as measured by ELISA after 24 h of CD40 ligation. The mean 6 SEM of three experiments performed with DCs or tonsillar B cells from different donors are shown.

IL12B expression levels in the virtual cell system with GM-CSF (CSF2) and IL4 over-expressed by 5 folds, CD40LG over-expressed by 10 folds and treated with p38-MAPK inhibitor (inhibiting the phosphorylation of P38-MAPK by around 75-80%) are compared with those in the virtual cell system with GM-CSF (CSF2) and IL4 over-expressed by 5 folds and CD40LG over-expressed by 10 folds.

Inference: Introduction of p38-MAPK inhibitor on CSF2 and IL4 differentiated, CD40LG stimulated dendritic cells leads to a decrease in IL12B levels.

15. Effect of CCL5 overexpression in CSF2 differentiated dendritic cells[16].

**EXPERIMENTAL**	**PREDICTIVE**

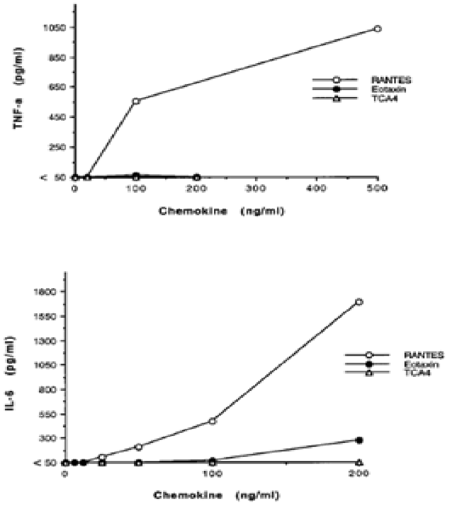

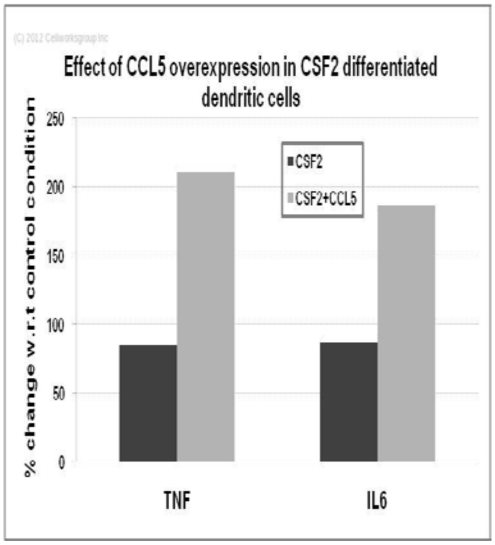

**Fig. 3B, C. RANTES induces cytokines.** DC (CSF2 differentiated) were treated with RANTES (100 ng/ml for 3 h) and then harvested for RNA preparation. Samples were tested by RPA. Comparisons of the ability of 100 ng/ml RANTES, eotaxin, and TCA4 to release TNF-a and IL-6 protein in 48-h conditioned medium by ELISA.	TNF and IL6 expression levels in the virtual cell system with GM-CSF (CSF2) over-expressed by 10 folds and CCL5 (RANTES) over-expressed by 100 are compared with those in the virtual cell system with GM-CSF over-expressed by 10 folds.
Inference: CCL5 over-expression in CSF2 differentiated dendritic cells leads to an increase in the levels of TNF and IL6	

References

1 Harvey, L. E. *et al.* Defensin DEFB103 bidirectionally regulates chemokine and cytokine responses to a pro-inflammatory stimulus. *Sci. Rep.* **3**, 1232, https://doi.org/10.1038/srep01232 (2013).

2 Borgwardt, D. S. *et al.* Histatin 5 binds to *Porphyromonas gingivalis* hemagglutinin B (HagB) and alters HagB-induced chemokine responses. *Sci. Rep.* https://doi.org/10.1038/srep03904 (2014).

3 Hegde, S., Pahne, J. & Smola-Hess, S. Novel immunosuppressive properties of interleukin-6 in dendritic cells: inhibition of NF-kappaB binding activity and CCR7 expression. *FASEB J.* **18**, 1439-1441, https://doi.org/10.1096/fj.03-0969fje (2004).

4 Aksoy, E., Amraoui, Z., Goriely, S., Goldman, M. & Willems, F. Critical role of protein kinase C epsilon for lipopolysaccharide-induced IL-12 synthesis in monocyte-derived dendritic cells. *Eur. J. Immunol.* **32**, 3040-3049, https://doi.org/10.1002/1521-4141(200211)32:11 (2002).

5 Melillo, J. A. *et al.* Dendritic cell (DC)-specific targeting reveals Stat3 as a negative regulator of DC function. *J. Immunol.* **184**, 2638-2645, https://doi.org/10.4049/jimmunol.0902960 (2010).

6 Yu, X. *et al.* Pattern recognition scavenger receptor CD204 attenuates Toll-like receptor 4-induced NF-kappaB activation by directly inhibiting ubiquitination of tumor necrosis factor (TNF) receptor-associated factor 6. *J. Biol. Chem.* **286**, 18795-18806, https://doi.org/10.1074/jbc.M111.224345 (2011).

7 Johnson, J. *et al.* Protein kinase Calpha is involved in interferon regulatory factor 3 activation and type I interferon-beta synthesis. *J Biol Chem* **282**, 15022-15032, https://doi.org/10.1074/jbc.M700421200 (2007).

8 Gaddis, D. E., Michalek, S. M. & Katz, J. Requirement of TLR4 and CD14 in dendritic cell activation by Hemagglutinin B from *Porphyromonas gingivalis*. *Mol. Immunol.* **46**, 2493-2504, https://doi.org/10.1016/j.molimm.2009.05.022 (2009).

9 Lin, Y. L., Liang, Y. C. & Chiang, B. L. Placental growth factor down-regulates type 1 T helper immune response by modulating the function of dendritic cells. *J. Leukoc. Biol.* **82**, 1473-1480, https://doi.org/10.1189/jlb.0307164 (2007).

10 Hackstein, H. *et al.* Rapamycin inhibits IL-4--induced dendritic cell maturation in vitro and dendritic cell mobilization and function in vivo. *Blood* **101**, 4457-4463, https://doi.org/10.1182/blood-2002-11-3370 (2003).

11 LeibundGut-Landmann, S. *et al.* Syk- and CARD9-dependent coupling of innate immunity to the induction of T helper cells that produce interleukin 17. *Nat. Immunol.* **8**, 630-638, https://doi.org/10.1038/ni1460 (2007).

12 Wesa, A. & Galy, A. Increased production of pro-inflammatory cytokines and enhanced T cell responses after activation of human dendritic cells with IL-1 and CD40 ligand. *BMC Immunol.* **3**, 14 (2002).

13 Salogni, L. *et al.* Activin A induces dendritic cell migration through the polarized release of CXC chemokine ligands 12 and 14. *Blood* **113**, 5848-5856, https://doi.org/10.1182/blood-2008-12-194597 (2009).

14 Dichmann, S. *et al.* Fractalkine induces chemotaxis and actin polymerization in human dendritic cells. *Inflamm. Res.* **50**, 529-533, https://doi.org/10.1007/PL00000230 (2001).

15 Aicher, A. *et al.* Differential role for p38 mitogen-activated protein kinase in regulating CD40-induced gene expression in dendritic cells and B cells. *J. Immunol.* **163**, 5786-5795 (1999).

16 Fischer, F. R., Luo, Y., Luo, M., Santambrogio, L. & Dorf, M. E. RANTES-induced chemokine cascade in dendritic cells. *J. Immunol.* **167**, 1637-1643, https://doi.org/10.4049/jimmunol.167.3.1637 (2001).

Supplementary Table S3. A Comparison of the observed reported responses (**a**) and predicted responses (**b**) of dendritic cells (DC) treated with LPS and pro-inflammatory agonists in 9 cocktail mixtures[1] validating the DC single cell computational model previously created[2,3].

a. Reported response of dendritic cells treated with LPS and pro-inflammatory agonists in 9 cocktail mixtures

Cocktail	Composition	Weak induction	Modest induction	Strong induction
LPS	LPS (0.1ug/ml)	IL-8, CCL2, CCL4, CXCL5, CXCL10, GM-CSF	IL-10, CCL20, CXCL1-3	IL-6, IL-12, TNFα, VEGF, CCL8, CCL15, CXCL1
1	IFNγ (20 ng/ml)+ TNFα (50 ng/ml)+ Poly I:C 12.5 ug/ml + IL-1β (10 ng/ml) + IFNα (6 ng/ml)	IL-8, CCL2, CCL4, CCL5, CXCL5, CXCL10	IL-10, CCL20, CXCL1-3, GM-CSF	IL-6, IL-12, TNFα, VEGF, CCL8, CCL15, CXCL1
2	IFNγ (20 ng/ml) + Poly I:C 12.5 ug/ml + IL-1β (10 ng/ml) + IFNα (6 ng/ml)	IL-8, CCL2, CCL4, CCL5, CXCL5, CXCL10	IL-10, CCL20, CXCL1-3, GM-CSF	IL-6, IL-12, TNFα, VEGF, CCL8, CCL15, CXCL1
3	IFNγ (20 ng/ml) + TNFα (50 ng/ml) + peptidoglycan (10 ug/ml)	IL-8, CCL2, CCL4, CCL5, CXCL5	IL-10, CCL20, CXCL1-3, CXCL10, GM-CSF	IL-6, IL-12, TNFα, VEGF, CCL8, CCL15, CXCL1
4	LPS (0.1 ug/ml) + IFNγ (20 ng/ml)	IL-8, CCL2, CCL4, CCL5, CXCL5, GM-CSF	IL-10, CCL20, CXCL1-3, CXCL10	IL-6, IL-12, TNFα, VEGF, CCL8, CCL15, CXCL1
5	LPS (0.1 ug/ml) + IFNγ (20 ng/ml) + TNFα (50 ng/ml)	IL-8, CCL2, CCL4, CCL5, CXCL5, GM-CSF	IL-10, CCL20, CXCL1-3, CXCL10	IL-6, IL-12, TNFα, VEGF, CCL8, CCL15, CXCL1
6	R848 (1.0 ug/ml)	IL-8, CCL2, CCL4, CCL5, CXCL10	IL-10, CCL15, CCL20, CXCL1-3, CXCL5, GM-CSF	IL-6, IL-12, TNFα, VEGF, CCL8, CXCL1

7	R848 (1.0 ug/ml) + IFNγ (20 ng/ml)	IL-8, CCL2, CCL4, CCL5, CCL20, CXCL5, GM-CSF	IL-10, CCL15, CCL20, CXCL1-3, CXCL10	IL-6, IL-12, TNFα, VEGF, CCL8, CXCL1
8	R848 (1,0 ug/ml) + poly I:C (10 ug/ml)	IL-8, CCL2, CCL4, CCL5, CXCL5, CXCL10	IL-10, CCL20, CXCL1-3, GM-CSF	IL-6, IL-12, TNFα, VEGF, CCL8, CCL15, CXCL1
9	IFNγ (20 ng/ml) + IL-1β (10 ng/ml)	IL-8, CCL2, CCL4, CCL5, CXCL5, GM-CSF	IL-10, CCL15, CCL20, CXCL1-3, CXCL10	IL-6, IL-12, TNFα, VEGF, CCL8, CXCL1

**b. Comparison of the observed reported and predicted responses of dendritic cells treated with LPS and pro-inflammatory agonists in 9 cocktail mixtures**																
**Cocktail**	**Composition**	**Bio-marker**	**Fold assayed**		**Trend analysis**	**Magnitude analysis**	**Bio-marker**	**Fold assayed**		**Trend analysis**	**Magnitude analysis**	**Bio-marker**	**Fold assayed**		**Trend analysis**	**Magnitude analysis**
**LPS**	**LPS**		**Experi-mental**	**Predic-tive**				**Experi-mental**	**Predic-tive**				**Experi-mental**	**Predic-tive**		
		CCL2	0-10	4.65	***Trend Match***	***Magnitude Match***	CXCL2	10-100	42.32	***Trend Match***	***Magnitude Match***	CXCL1	>100	3648.62	***Trend Match***	***Magnitude Match***
		CSF2	0-10	4.39	***Trend Match***	***Magnitude Match***	IL10	10-100	24.38	***Trend Match***	***Magnitude Match***	IL12A	>100	127.85	***Trend Match***	***Magnitude Match***
		IL8	0-10	7.37	***Trend Match***	***Magnitude Match***	CCL20	10-100	49.74	***Trend Match***	***Magnitude Match***	IL6	>100	369.86	***Trend Match***	***Magnitude Match***
		CCL4	0-10	6.96	***Trend Match***	***Magnitude Match***						IL12B	>100	1010.44	***Trend Match***	***Magnitude Match***
		CXCL10	0-10	4.26	***Trend Match***	***Magnitude Match***						VEGFA	>100	516.44	***Trend Match***	***Magnitude Match***
												TNF	>100	266.86	***Trend Match***	***Magnitude Match***
**1**	**IFNG + TNF + IFNA1 + IL1B + PolyIC**	CCL2	0-10	4.44	***Trend Match***	***Magnitude Match***	CSF2	10-100	23.26	***Trend Match***	***Magnitude Match***	CXCL1	>100	2094.90	***Trend Match***	***Magnitude Match***
		IL8	0-10	7.02	***Trend Match***	***Magnitude Match***	IL10	10-100	16.92	***Trend Match***	***Magnitude Match***	IL12A	>100	109.47	***Trend Match***	***Magnitude Match***
		CCL4	0-10	5.28	***Trend Match***	***Magnitude Match***	CCL20	10-100	32.76	***Trend Match***	***Magnitude Match***	IL6	>100	283.78	***Trend Match***	***Magnitude Match***

		CXCL10	0-10	9.57	***Trend Match***	***Magnitude Match***	CXCL2	10-100	29.87	***Trend Match***	***Magnitude Match***	IL12B	>100	820.15	***Trend Match***	***Magnitude Match***
		CCL5	0-10	4.97	***Trend Match***	***Magnitude Match***						VEGFA	>100	333.72	***Trend Match***	***Magnitude Match***
												TNF	>100	221.78	***Trend Match***	***Magnitude Match***
**2**	**IFNG + IFNA1 + IL1B + PolyIC**	CCL2	0-10	4.07	***Trend Match***	***Magnitude Match***	IL10	10-100	13.51	***Trend Match***	***Magnitude Match***	CXCL1	>100	414.39	***Trend Match***	***Magnitude Match***
		IL8	0-10	5.26	***Trend Match***	***Magnitude Match***	CSF2	10-100	18.31	***Trend Match***	***Magnitude Match***	IL12A	>100	100.19	***Trend Match***	***Magnitude Match***
		CCL4	0-10	4.58	***Trend Match***	***Magnitude Match***	CXCL2	10-100	24.00	***Trend Match***	***Magnitude Match***	IL6	>100	231.18	***Trend Match***	***Magnitude Match***
		CXCL10	0-10	8.52	***Trend Match***	***Magnitude Match***	CCL20	10-100	26.63	***Trend Match***	***Magnitude Match***	IL12B	>100	737.70	***Trend Match***	***Magnitude Match***
		CCL5	0-10	4.01	***Trend Match***	***Magnitude Match***						VEGFA	>100	270.74	***Trend Match***	***Magnitude Match***
												TNF	>100	198.32	***Trend Match***	***Magnitude Match***
**3**	**IFNG + TNF + peptidoglycan**	CCL2	0-10	5.50	***Trend Match***	***Magnitude Match***	CCL20	10-100	37.00	***Trend Match***	***Magnitude Match***	CXCL1	>100	2703.68	***Trend Match***	***Magnitude Match***
		CCL4	0-10	7.43	***Trend Match***	***Magnitude Match***	CSF2	10-100	28.74	***Trend Match***	***Magnitude Match***	IL12A	>100	115.45	***Trend Match***	***Magnitude Match***
		CCL5	0-10	6.98	***Trend Match***	***Magnitude Match***	IL10	10-100	18.96	***Trend Match***	***Magnitude Match***	IL12B	>100	907.77	***Trend Match***	***Magnitude Match***
		IL8	0-10	7.36	***Trend Match***	***Magnitude Match***	CXCL10	10-100	67.21	***Trend Match***	***Magnitude Match***	IL6	>100	290.24	***Trend Match***	***Magnitude Match***
							CXCL2	10-100	38.25	***Trend Match***	***Magnitude Match***	TNF	>100	234.90	***Trend Match***	***Magnitude Match***
												VEGFA	>100	379.12	***Trend Match***	***Magnitude Match***
**4**	**IFNG + LPS**	CCL2	0-10	4.91	***Trend Match***	***Magnitude Match***	CXCL2	10-100	46.21	***Trend Match***	***Magnitude Match***	CXCL1	>100	3017.94	***Trend Match***	***Magnitude Match***
		CSF2	0-10	3.18	***Trend Match***	***Magnitude Match***	CXCL10	10-100	12.53	***Trend Match***	***Magnitude Match***	IL12A	>100	130.59	***Trend Match***	***Magnitude Match***

		IL8	0-10	7.28	***Trend Match***	***Magnitude Match***	IL10	10-100	24.49	***Trend Match***	***Magnitude Match***	IL6	>100	389.84	***Trend Match***	***Magnitude Match***
		CCL4	0-10	7.33	***Trend Match***	***Magnitude Match***	CCL20	10-100	53.53	***Trend Match***	***Magnitude Match***	IL12B	>100	1036.96	***Trend Match***	***Magnitude Match***
		CCL5	0-10	5.73	***Trend Match***	***Magnitude Match***						VEGFA	>100	556.30	***Trend Match***	***Magnitude Match***
												TNF	>100	273.79	***Trend Match***	***Magnitude Match***
**5**	**IFNG + LPS + TNF**	CSF2	0-10	3.73	***Trend Match***	***Magnitude Match***	CXCL2	10-100	52.59	***Trend Match***	***Magnitude Match***	CXCL1	>100	4033.90	***Trend Match***	***Magnitude Match***
		CCL2	0-10	5.11	***Trend Match***	***Magnitude Match***	IL10	10-100	27.16	***Trend Match***	***Magnitude Match***	IL12A	>100	134.81	***Trend Match***	***Magnitude Match***
		IL8	0-10	8.57	***Trend Match***	***Magnitude Match***	CCL20	10-100	60.27	***Trend Match***	***Magnitude Match***	IL6	>100	428.56	***Trend Match***	***Magnitude Match***
		CCL4	0-10	7.74	***Trend Match***	***Magnitude Match***	CXCL10	10-100	11.01	***Trend Match***	***Magnitude Match***	IL12B	>100	1074.65	***Trend Match***	***Magnitude Match***
		CCL5	0-10	6.72	***Trend Match***	***Magnitude Match***						VEGFA	>100	623.55	***Trend Match***	***Magnitude Match***
												TNF	>100	286.15	***Trend Match***	***Magnitude Match***
**6**	**R848**	CCL2	0-10	4.41	***Trend Match***	***Magnitude Match***	CSF2	10-100	11.12	***Trend Match***	***Magnitude Match***	CXCL1	>100	176.42	***Trend Match***	***Magnitude Match***
		IL8	0-10	8.72	***Trend Match***	***Magnitude Match***	CXCL2	10-100	29.55	***Trend Match***	***Magnitude Match***	IL12A	>100	103.86	***Trend Match***	***Magnitude Match***
		CCL4	0-10	5.14	***Trend Match***	***Magnitude Match***	IL10	10-100	18.96	***Trend Match***	***Magnitude Match***	IL6	>100	265.72	***Trend Match***	***Magnitude Match***
		CXCL10	0-10	2.18	***Trend Match***	***Magnitude Match***	CCL20	10-100	32.01	***Trend Match***	***Magnitude Match***	IL12B	>100	784.25	***Trend Match***	***Magnitude Match***
		CCL5	0-10	6.00	***Trend Match***	***Magnitude Match***						VEGFA	>100	314.01	***Trend Match***	***Magnitude Match***
												TNF	>100	210.92	***Trend Match***	***Magnitude Match***
**7**	**IFNG + R848**	CCL2	0-10	5.79	***Trend Match***	***Magnitude Match***	CXCL2	10-100	68.60	***Trend Match***	***Magnitude Match***	CXCL1	>100	762.64	***Trend Match***	***Magnitude Match***
		CSF2	0-10	4.14	***Trend Match***	***Magnitude Match***	CXCL10	10-100	32.16	***Trend Match***	***Magnitude Match***	IL12A	>100	140.98	***Trend Match***	***Magnitude Match***

		CCL20	0-10	74.06	***Trend Match***	***Magnitude Mismatch***	IL10	10-100	30.55	***Trend Match***	***Magnitude Match***	IL6	>100	526.18	***Trend Match***	***Magnitude Match***
		IL8	0-10	8.48	***Trend Match***	***Magnitude Match***						IL12B	>100	1136.95	***Trend Match***	***Magnitude Match***
		CCL4	0-10	8.16	***Trend Match***	***Magnitude Match***						VEGFA	>100	765.44	***Trend Match***	***Magnitude Match***
		CCL5	0-10	8.08	***Trend Match***	***Magnitude Match***						TNF	>100	310.94	***Trend Match***	***Magnitude Match***
**8**	**PolyIC + R848**	CCL2	0-10	5.32	***Trend Match***	***Magnitude Match***	CXCL2	10-100	65.64	***Trend Match***	***Magnitude Match***	CXCL1	>100	1009.00	***Trend Match***	***Magnitude Match***
		IL8	0-10	8.19	***Trend Match***	***Magnitude Match***	CSF2	10-100	43.25	***Trend Match***	***Magnitude Match***	IL12A	>100	142.71	***Trend Match***	***Magnitude Match***
		CCL4	0-10	7.85	***Trend Match***	***Magnitude Match***	IL10	10-100	31.36	***Trend Match***	***Magnitude Match***	IL6	>100	545.57	***Trend Match***	***Magnitude Match***
		CXCL10	0-10	4.04	***Trend Match***	***Magnitude Match***	CCL20	10-100	75.80	***Trend Match***	***Magnitude Match***	IL12B	>100	1139.02	***Trend Match***	***Magnitude Match***
		CCL5	0-10	6.99	***Trend Match***	***Magnitude Match***						VEGFA	>100	785.46	***Trend Match***	***Magnitude Match***
												TNF	>100	315.68	***Trend Match***	***Magnitude Match***
**9**	**IFNG + IL1B**	CCL2	0-10	5.69	***Trend Match***	***Magnitude Match***	CXCL2	10-100	40.07	***Trend Match***	***Magnitude Match***	CXCL1	>100	433.42	***Trend Match***	***Magnitude Match***
		IL8	0-10	2.57	***Trend Match***	***Magnitude Match***	CXCL10	10-100	89.35	***Trend Match***	***Magnitude Match***	IL12A	>100	115.66	***Trend Match***	***Magnitude Match***
		CCL4	0-10	5.92	***Trend Match***	***Magnitude Match***	IL10	10-100	18.96	***Trend Match***	***Magnitude Match***	IL6	>100	297.64	***Trend Match***	***Magnitude Match***
		CXCL10	0-10	7.16	***Trend Match***	***Magnitude Match***	CCL20	10-100	38.70	***Trend Match***	***Magnitude Match***	IL12B	>100	920.32	***Trend Match***	***Magnitude Match***
		CCL5	0-10	6.95	***Trend Match***	***Magnitude Match***						VEGFA	>100	399.09	***Trend Match***	***Magnitude Match***
												TNF	>100	235.94	***Trend Match***	***Magnitude Match***

References

1 Jensen, S. S. & Gad, M. Differential induction of inflammatory cytokines by dendritic cells treated with novel TLR-agonist and cytokine based cocktails: targeting dendritic cells in autoimmunity. *J. Inflamm. (Lond).* **7**, 37, https://doi.org/10.1186/1476-9255-7-37 (2010).

2 Harvey, L. E. *et al.* Defensin DEFB103 bidirectionally regulates chemokine and cytokine responses to a pro-inflammatory stimulus. *Sci. Rep.* **3**, 1232, https://doi.org/10.1038/srep01232 (2013).

3 Borgwardt, D. S. *et al.* Histatin 5 binds to *Porphyromonas gingivalis* hemagglutinin B (HagB) and alters HagB-induced chemokine responses. *Sci. Rep.* **4**, 3904, https://doi.org/10.1038/srep03904 (2014).

Supplementary Table S4. Comparison of the predicted responses from the single cell computational models (**a**) and the multi-cell computational models (**b**) of gingival epithelial keratinocytes (GE KER), dendritic cells (DC), and helper T lymphocytes (HTL) with the observed responses from the single cell cultures (**a**) and multi-cell cultures (**b**) when treated with LPS and measured at 64 hours.

a. All three models showed 100%, 80%, and 90% correlation in trend (e.g. direction of change) with the observed analyte concentrations from DC, GE KER, and HTL, respectively.

Biomarker	DC (10 μg/ml LPS)			GE KER (10 μg/ml LPS)			HTL (10 μg/ml LPS)		
	Pred.*	Exp.* (mean)	Trend**	Predicted*	Exp.* (mean)	Trend**	Pred.*	Exp.* (mean)	Trend**
CCL3	276.03	207.63	MATCH	258.55	87.74	MATCH	172.84	172.96	MATCH
CCL4	395.33	648.45	MATCH	-11.29	-49.14	MATCH	430.06	91.34	MATCH
CCL5	251.46	102.56	MATCH	2003.89	-7.62	MISMATCH	236.79	50.26	MATCH
CSF2	453.08	17356.20	MATCH	248.84	69.51	MATCH	80.75	30.80	MATCH
IL12B	358.28	787.15	MATCH	290.62	-2.81	MISMATCH	370.60	59.17	MATCH
IL1A	358.08	41.78	MATCH	273.22	78.84	MATCH	580.97	103.06	MATCH
IL6	291.25	7639.55	MATCH	280.97	425.16	MATCH	126.61	45.26	MATCH
IL8	268.61	72.90	MATCH	474.37	105.05	MATCH	502.55	291.93	MATCH
TNF	806.39	10045.85	MATCH	908.31	28.25	MATCH	117.31	366.55	MATCH
VEGFA	1014.35	26.03	MATCH	308.26	24.39	MATCH	339.36	-14.61	MISMATCH
		**Match %**	**100%**		**Match %**	**80%**		**Match %**	**90%**

*Percent change with respect to control
**Represents the match (or mismatch) between experimental versus predictive trend (e.g. direction of increase/decrease)

b. All three multi-cell models showed 80% correlation in trend (e.g. direction of change) with the respective observed analyte concentrations.

Marker	GE KER + DC			GE KER+ HTL			DC + HTL		
	Pred.*	Exp.* (mean)	Trend**	Pred.*	Exp.* (mean)	Trend**	Pred.*	Exp.* (mean)	Trend**
CCL3	180.30	101.15	MATCH	126.59	183.96	MATCH	95.88	73.82	MATCH
CCL4	103.03	52.23	MATCH	103.03	497.73	MATCH	131.00	40.55	MATCH
CCL5	213.07	6.27	MATCH	177.44	7.52	MATCH	90.06	1.24	MISMATCH
CSF2	213.91	22.84	MATCH	142.93	5.33	MATCH	122.78	75.48	MATCH
IL12(p40)	192.89	-32.47	MISMATCH	165.37	-6.71	MISMATCH	131.13	181.33	MATCH
IL1A	206.09	8.49	MATCH	195.62	41.32	MATCH	135.60	1.61	MISMATCH
IL6	164.81	20.00	MATCH	142.06	146.51	MATCH	61.65	49.54	MATCH
IL8	241.22	208.16	MATCH	217.65	70.24	MATCH	102.76	198.64	MATCH
TNFA	455.41	48.90	MATCH	343.81	27.19	MATCH	146.64	53.62	MATCH
VEGFA	238.98	-32.49	MISMATCH	167.02	-36.13	MISMATCH	122.73	25.89	MATCH
		**Match %**	**80%**		**Match %**	**80%**		**Match %**	**80%**
